# In Vitro Metabolism of Six C-Glycosidic Flavonoids from *Passiflora incarnata* L.

**DOI:** 10.3390/ijms22126566

**Published:** 2021-06-18

**Authors:** Martina Tremmel, Josef Kiermaier, Jörg Heilmann

**Affiliations:** 1 Department of Pharmaceutical Biology, Faculty of Chemistry and Pharmacy, University of Regensburg, Universitätsstr. 31, 93053 Regensburg, Germany; martina.tremmel@chemie.uni-regensburg.de; 2 Department of Central Analytics, Faculty of Chemistry and Pharmacy, University of Regensburg, Universitätsstr. 31, 93053 Regensburg, Germany; josef.kiermaier@ur.de

**Keywords:** flavonoids, metabolism, C-glycosides, C–C cleavage, Caco-2 cells, *Passiflora incarnata* L.

## Abstract

Several medical plants, such as *Passiflora incarnata* L., contain C-glycosylated flavonoids, which may contribute to their efficacy. Information regarding the bioavailability and metabolism of these compounds is essential, but not sufficiently available. Therefore, the metabolism of the C-glycosylated flavones orientin, isoorientin, schaftoside, isoschaftoside, vitexin, and isovitexin was investigated using the Caco-2 cell line as an in vitro intestinal and epithelial metabolism model. Isovitexin, orientin, and isoorientin showed broad ranges of phase I and II metabolites containing hydroxylated, methoxylated, and sulfated compounds, whereas schaftoside, isoschaftoside, and vitexin underwent poor metabolism. All metabolites were identified via UHPLC-MS or UHPLC-MS/MS using compound libraries containing all conceivable metabolites. Some structures were confirmed via UHPLC-MS experiments with reference compounds after a cleavage reaction using glucuronidase and sulfatase. Of particular interest is the observed cleavage of the C–C bonds between sugar and aglycone residues in isovitexin, orientin, and isoorientin, resulting in unexpected glucuronidated or sulfated luteolin and apigenin derivatives. These findings indicate that C-glycosidic flavones can be highly metabolized in the intestine. In particular, flavonoids with ortho-dihydroxy groups showed sulfated metabolites. The identified glucuronidated or sulfated aglycones demonstrate that enzymes expressed by Caco-2 cells are able to potentially cleave C–C bonds in vitro.

## 1. Introduction

*Passiflora incarnata* L. is a medical plant used for the treatment of restlessness, insomnia, anxiety, and nervous stress [1]. Flavonoids are the most abundant class of secondary natural compounds in *P. incarnata*, representing up to 2.5% of the dried plant; they are potentially co-responsible for the plant’s pharmacological effects [2,3,4], and possibly have a prodrug effect requiring metabolic activation [5]. Many naturally found flavonoids are glycosides, varying in sugar moiety and binding type. Most of them exist as *O-*glycosides, and the sugar is linked to the aglycone via an *O-*glycosidic bond. In contrast, the predominant flavonoids found in the aerial parts of *P. incarnata* L. are C-glycosidic flavonoids, showing a C–C glycosidic bond between the sugar and the C-8 or C-6 positions of the aglycones apigenin and luteolin [6]. Despite the fact that they are less well investigated than *O-*glycosidic flavonoids, C-glycosidic flavonoids show anticancer [7], antiviral [8], antidiabetic [9], and hepatoprotective activities [10]. Thus, positive effects on human health are expected, but unexplored.

Extensive investigations of the metabolism of *O-*glycosidic flavonoids have revealed that they are usually absorbed as aglycones after deglycosidation, followed by rapid phase I and II metabolism during passage through the intestinal epithelium and the liver. The initial metabolism step proceeds via hydrolysation in the small intestine by lactase-phlorizin hydrolase to the corresponding aglycones, which are absorbed and metabolized in phase I and II reactions to various *O-*glucuronides and *O-*sulfates. Specifically, enzymes such as cytochrome P450s (CYPs) [11,12], catechol-O-methyltransferases (COMTs) [13,14], uridine 5′-diphospho glucuronosyltransferases (UGTs) [15], and sulfotransferases (SULTs) [16,17] are involved. In addition, disposal through efflux transporters has been described [18]. Alternatively, degradation of the aglycones to smaller phenolic metabolites and resorption or excretion with the feces is also possible [19]. Compared to *O-*glycosides, C-glycosides are hydrolytically more stable. Therefore, deglycosylation to their corresponding aglycones by intestinal hydrolytic enzymes, which easily cleave the C–O bonds of *O-*glycosides, is unlikely. Thus, *O-* and C-glycosides likely differ in their pharmacokinetic behavior; however, data on the metabolism of C-glycosides are scarce.

Results on the metabolism of C-glycosides are still lacking, as the metabolic profiles of C-glycosides have not been published to date. Nevertheless, incubation of apigenin C-glycosides with human fecal samples has indicated that they can be deglycosylated in the colon by human intestinal bacteria to release the corresponding aglycones. This is followed by partial cleavage to smaller phenolic metabolites and excretion in the feces or absorption in the colon. Nevertheless, a significant amount of C-glycosides may remain intact in the small intestine, and can be absorbed, distributed, and eliminated [20].

**Figure 1:**
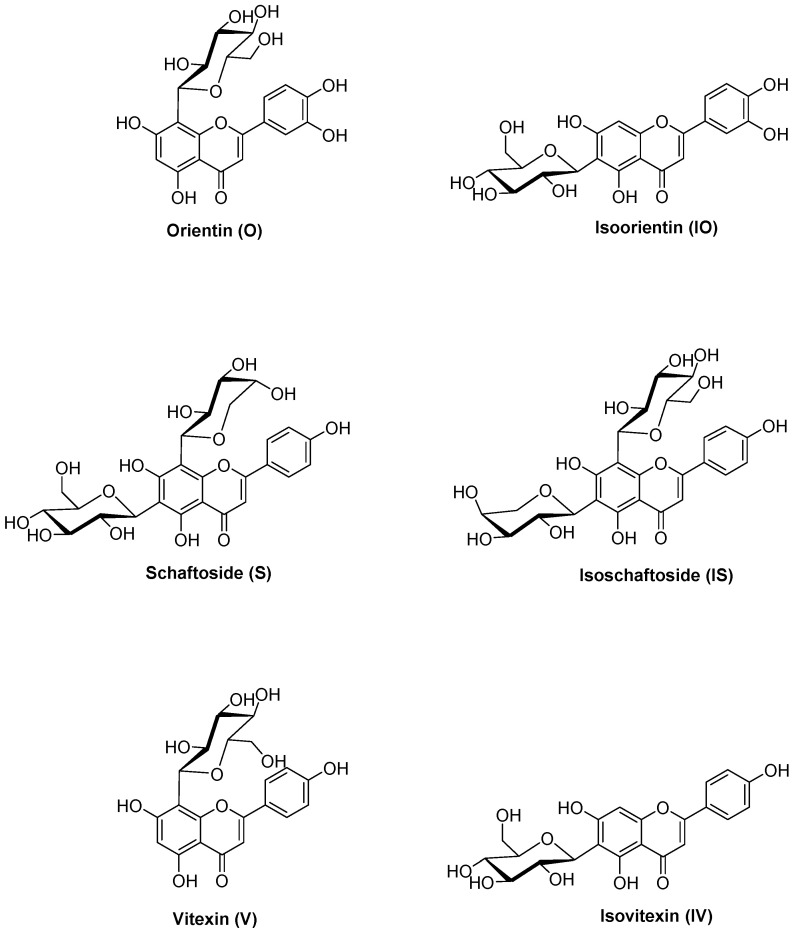
Structures of the C-glycosidic flavonoids orientin (O), isoorientin (IO), schaftoside (S), isoschaftoside (IS), vitexin (V), and isovitexin (IV).

Orientin (luteolin 8-C-glucoside), isoorientin (luteolin 6-C-glucoside), schaftoside (apigenin 6-C-glucoside-8-C-arabinoside), isoschaftoside (apigenin 8-C-glucoside-6-C-arabinoside), vitexin (apigenin 8-C-glucoside), and isovitexin (apigenin 6-C-glucoside) are six representative C-glycosidic flavonoids present in *P. incarnata* L. [21] (Figure 1). Orientin and isoorientin have shown various pharmacological in vitro and in vivo properties [22], such as antioxidative [23], antiviral [24], anti-inflammatory [25,26,27], antidepressant-like [28], and antinociceptive effects [29]. While vitexin also exhibits antioxidative [30,31], antinociceptive [32,33], anti-inflammatory [34,35], antimicrobial [36,37], and antiviral effects [38], isovitexin has been poorly studied, but seems to have a similar pharmacological profile [39]. However, the pharmacological effects of schaftoside and isoschaftoside have not yet been studied [40].

Although poor direct absorption of C-glycosidic flavonoids is expected [41], they could still show efficacy, as the metabolism of absorbed C-glycosides has rarely been explored.

The aim of this study was to identify possible phase I and II metabolites of C-glycosidic flavonoids by investigating orientin (O), isoorientin (IO), schaftoside (S), isoschaftoside (IS), vitexin (V), and isovitexin (IV) (Figure 1), using an in vitro Caco-2 cell monolayer model. The objective was to enhance our understanding of metabolism in vitro and in vivo, and to provide relevant information as a basis for further studies.

## 2. Results

### 2.1. Stability Testing

To guarantee the stability of all test substances during the metabolism assay on Caco-2 cells, they were incubated in the test buffer under assay conditions. Test samples were collected each hour for 5 h and were measured via HPLC-DAD. All tested substances showed high levels of stability under assay conditions. After 3 h of incubation, O, IO, S, IS, and V showed values of approximately 100%, and only the content of IV was reduced to 96%. Nearly identical results were detected after a 5 h incubation time, where a content of 95% could be demonstrated for IV. Statistically, stability values did not differ from the 100% value (*p* < 0.05). Considering the standard deviations, all measured substances showed sufficient stability for metabolism testing up to 5 h. The determined values of the stability testing from all six C-glycosidic flavonoids are shown in Figure S1.

### 2.2. Cell Viability

Cell viability was evaluated using an MTT assay under conditions comparable to the metabolism approach. All six substances were tested in five different dilutions in HBSS (pH 6.0). The determined viability values of O, IO, S, IS, V, and IV are listed in the Supplementary Materials (Figure S2). In summary, O, IO, S, and IS showed no significant effects at any concentration on the viability of Caco-2 cells, while V and IV showed slight, concentration-dependent reductions (V: 83% viability at 100 µM, 92% at 10 µM; IV: 82% viability at 100 µM, 92% at 10 µM). Statistically, viability values did not differ significantly from the 100% value (*p* < 0.05). Overall, none of the six C-glycosidic flavonoids had a decisive effect on the viability of Caco-2 cells under these conditions.

### 2.3. Metabolite Profiles of C-Glycosidic Flavonoids

**Figure 2:**
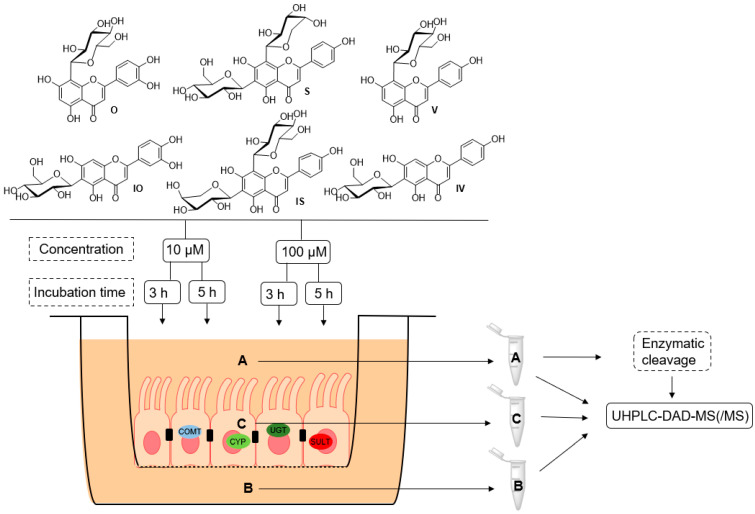
Schematic overview of the metabolic experiments on orientin (O), isoorientin (IO), schaftoside (S), isoschaftoside (IS), vitexin (V), and isovitexin (IV), with different incubation times and concentrations, using the Caco-2 monolayer model; A: apical, B: basolateral, C: cell lysate, COMT: catechol-O-methyltransferase, UGT: UDP-glucuronosyltransferase, SULT: sulfotransferase, CYP: cytochrome P450.

**Figure 3:**
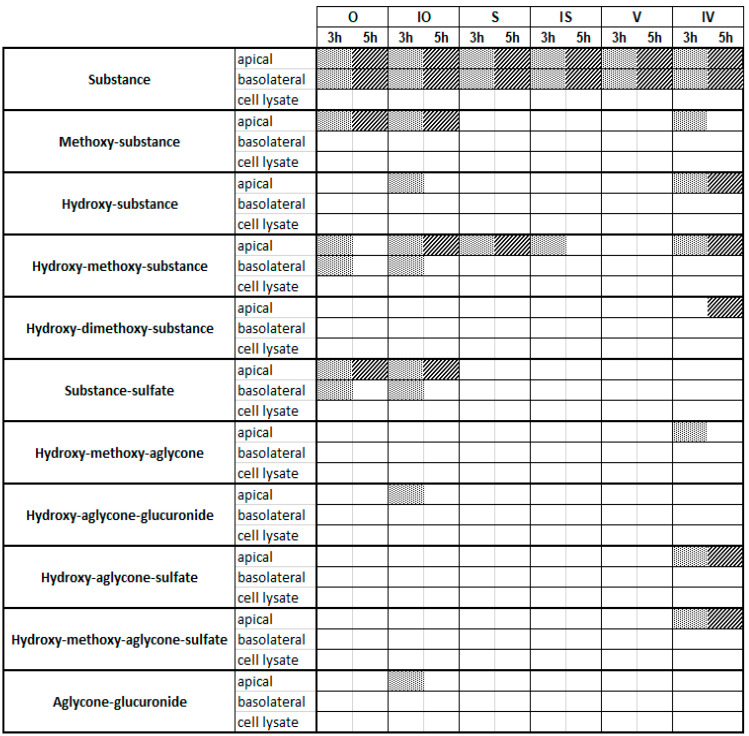
Metabolites detected via UHPLC-MS after a Caco-2 metabolism experiment with 10 µM of the testing substances and incubation times of 3 h and 5 h. Dot line and diagonal line are used for better visualization meaning that the metabolite is detectable.

**Figure 4:**
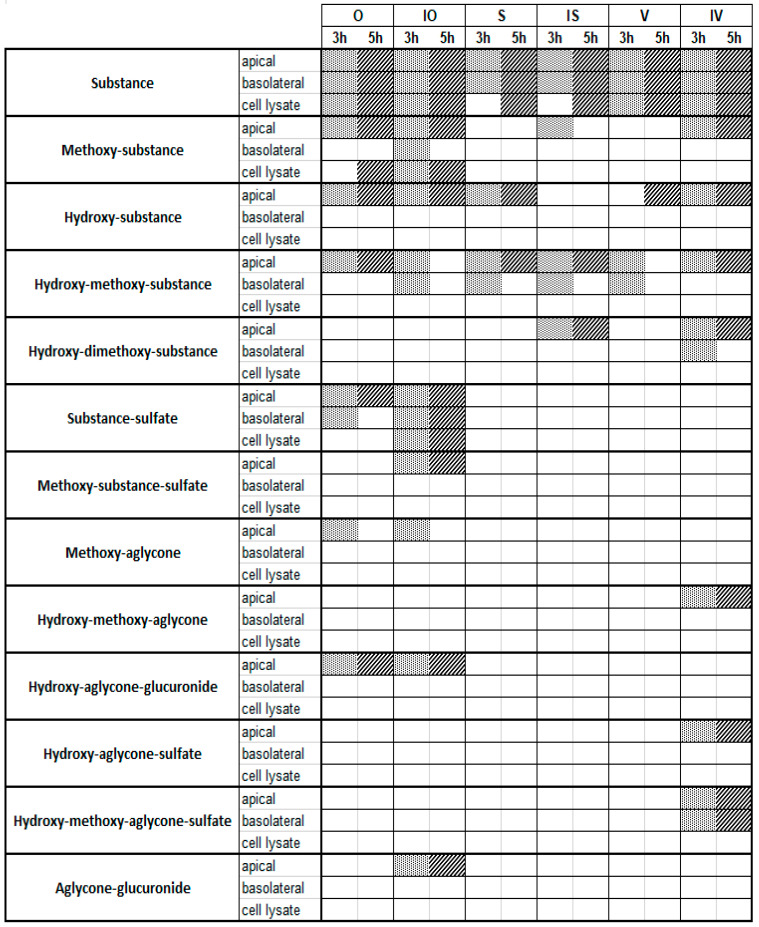
Metabolites detected via UHPLC-MS after a Caco-2 metabolism experiment with 100 µM of the testing substances and incubation times of 3 h and 5 h. Dot line and diagonal line are used for better visualization meaning that the metabolite is detectable.

To obtain information on the in vitro metabolism of the six tested C-glycosidic flavonoids in the intestine, experiments were carried out using the Caco-2 monolayer model (Figure 2). Metabolite profiles were characterized at two different concentrations (10 and 100 µM; *n* = 3) and after two different incubation times (3 or 5 h; *n* = 3) in order to determine the influence of concentration and time on the metabolism reactions that occurred. Furthermore, to analyze the distribution of the observed metabolites, samples were collected from the cell lysate and the apical and basolateral compartments. All samples of the metabolism experiments using the Caco-2 cell monolayer were analyzed using UHPLC-DAD-MS and UHPLC-DAD-MS/MS. Metabolites were identified by libraries that were prepared for each compound (Tables S1–S6). The O and IO libraries contained 67 metabolites (Tables S1 and S2), the S and IS libraries contained 106 metabolites (Tables S3 and S4), and the V and IV libraries contained 71 possible metabolites (Tables S5 and S6). Overviews of the metabolites found at 10 and 100 µM are shown in Figure 3 and Figure 4, respectively (see also Tables S7–S27). Metabolic artefacts due to assay conditions or cytotoxicity of the test compounds can be excluded because of the previously performed stability testing and cell viability assay (see Figures S1 and S2).

At a concentration of 10 µM, it became obvious that the six C-glycosides underwent different metabolism pathways (Figure 3): V, S, and IS showed very limited metabolism, and only hydroxylated/methoxylated C-glycosides could be observed as metabolites. No phase II conjugates containing glucuronic acid or sulfates were detectable. In contrast, O, IO, and IV showed broader ranges of phase I and II metabolites. As phase I products, mainly hydroxylated/methoxylated flavones could be found, whereas sulfates dominated as conjugation products. By comparison of the incubation times (3 and 5 h), it became obvious that the number of hydroxylated metabolites increased with time, while methylated metabolites decreased. Interestingly, at 10 µM, nearly all metabolites except for a very small number of metabolites of O and IO could be found only in the apical compartment, which was probably caused by efflux transporters, and covered all six C-glycosides [18]. In contrast, the original test substances were all present in the apical and basolateral compartments, suggesting slow but detectable penetration of all six C-glycosides through the cells.

**Figure 5:**
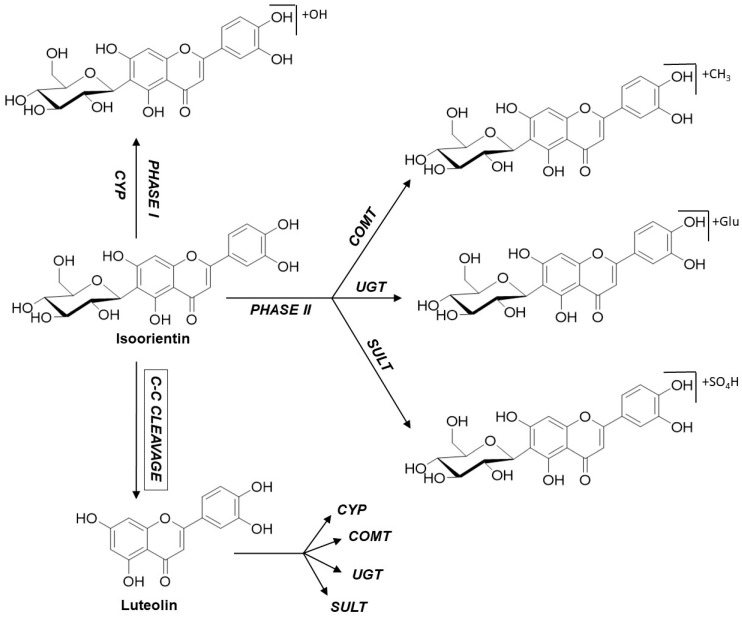
Metabolism pathways shown for the example of isoorientin.

In line with these observations, the 100 µM approach also showed a few hydroxylated and methoxylated derivatives for V, S, and IS after 3 and 5 h (Figure 4). The diversity of the metabolite profiles was again low, as all three compounds still lacked the presence of phase II metabolites with glucuronic acid or sulfates as the conjugation substrate. In contrast, O, IO, and IV showed extensive metabolism, with increasing metabolite diversity compared to the 10 µM approach mentioned above. Various hydroxylated and methoxylated metabolites, as well as phase II conjugates of the initial substances with sulfates and glucuronic acid, were detected. In particular, O and IO methylated, hydroxylated, and even several sulfated monoconjugates were detected. O and IO were the only substances containing an ortho-dihydroxy structure at C-3′/C-4′ in the B ring, which could be a favorable structural feature for the conversion of C-glycosylated flavones by the sulfotransferases expressed in Caco-2 cells. Surprisingly, metabolites of the corresponding aglycones apigenin and luteolin were also detected for O, IO, and IV, pointing to cleavage of the C–C glycosidic bond (Figure 5). Moreover, in the case of IV, conjugation with sulfate was detected solely after C–C cleavage of the glycosidic bond. Notably, the preferred sulfation of O and IO could be explained by the presence of an ortho-dihydroxy structure at C-3′/C-4′ in the B rings of the respective flavones. In comparison to the 10 µM approach, at an initial concentration of 100 µM, C-glycosides could be found not only in the apical and basolateral compartments, but also in the cell lysate. The presence of metabolites in the basolateral compartment increased slightly, but there were still more metabolites in the apical compartment. The presence of metabolites in cell lysates is a very exceptional case and, again, these findings suggest that efflux transporters accept the formed metabolites as substrates [18].

Notably, sulfated and glucuronidated conjugates of the corresponding aglycones of O, IO, and IV—apigenin and luteolin—could be detected. In the case of IV, this type of metabolite even provided the predominant conjugates. Some of these structures could be completely confirmed by UHPLC-MS experiments with reference compounds after a cleavage reaction using glucuronidase and sulfatase (Table 1). Thus, the present results demonstrate that enzymes expressed by Caco-2 cells potentially cleave C–C bonds in vitro, which has not yet been described, while bacterial enzymes of the intestinal microflora have been proven to cleave C–C bonds, this has not been described for human intestinal epithelium cells.

## 3. Discussion

Information on the in vitro and in vivo metabolism of C-glycosidic flavonoids is quite limited. Single investigations of some apigenin C-glycosides have addressed their metabolism with human fecal samples and, thus, with the metabolic activity of human intestinal bacteria [20]. Thus, a broader investigation of their metabolism, which has already been performed for *O-*glycosidic flavonoids [16,17,18], is necessary for C-glycosidic flavones. Therefore, we investigated the metabolism of six C-flavones in the established in vitro Caco-2 cell monolayer model, in order to obtain further information on their potential intestinal metabolism profiles as a function of incubation time and concentration. As test substances, six flavonoids with luteolin or apigenin basic structures linked to one or two sugar residues were used. As these compounds differ only in their number of sugar moieties and/or hydroxy groups in the B-ring, structure–metabolism relationships should be investigated. In addition, they often occur as secondary metabolites of medicinal plants of the genera *Passiflora*, *Viola*, and *Crataegus*.

Independent of incubation time and concentration, three of the test compounds—schaftoside, isoschaftoside, and vitexin—are poorly metabolized in phase I and II reactions, while the other three—orientin, isoorientin, and isovitexin—seem to be highly metabolized in phase I and II reactions. For the identification of simple structural elements as a prerequisite for varying metabolisms, the compound set is too small, and requires expansion in further studies. However, it can be proposed that the dihydroxy structure at C-3′/C-4′ is the meaningful structural feature for the conversion by sulfotransferases in Caco-2 cells, as only sulfated conjugates of O and IO could be detected.

It is also notable that sulfates were the predominant phase II conjugates, while only small amounts of glucuronides could be identified. In the literature, some studies on polyphenols accordingly report on the influence of the initial test concentration on the metabolite profile. For example, it has been described for resveratrol that low initial concentrations mainly favored sulfation through SULTs, while at higher concentrations, glucuronidation has also been observed [42]. Here, the inserted compound concentration increased the metabolite profile, but a shift from sulfated metabolites to glucuronidated conjugates was not detected. One possible explanation is that the efflux activity of transporters still ensures relatively low concentrations in the cells.

Furthermore, mechanistically relevant questions with regard to transport behavior in vitro and in vivo must also be investigated more deeply for C-glycosides of flavonoids. Most of the detected metabolites were found in the apical compartment, which simulates the intestine. A possible explanation for this is efflux transporters, which are known to transport many kinds of different flavonoids out of intestinal cells [18]. This means that a high number of metabolites are probably transported out of the cell interior, where they were formed by phase I and phase II enzymes. In the intestine, these metabolites will be substrates of the bacterial microflora, allowing them to form further metabolites.

Moreover, it was investigated whether enzymes expressed by Caco-2 cells could potentially cleave C–C bonds in vitro. For O, IO, and especially IV, metabolites of the corresponding aglycones luteolin and apigenin could also be identified. These metabolites contain sulfated, glucuronidated, methylated, and hydroxylated conjugates of the aglycones, demonstrating that enzymes expressed not only by intestinal bacteria but also by mammalian cells are able to potentially cleave C–C bonds in vitro.

The detected phase I and II metabolite profiles build an objective to enhance our understanding of metabolism. The limited number of metabolites in the compound libraries results in a lack of identification of some metabolites. Nevertheless, the detected phase I and II metabolites of the initial substances, as well as the metabolites of the corresponding aglycones, build an objective to enhance our understanding of metabolism in vivo, and to provide relevant information as a basis for further studies. In particular, the fact that enzymes expressed by Caco-2 cells could potentially cleave C–C bonds should be further investigated. C-glycosidic flavonoids most likely undergo different metabolic steps, resulting in a very broad in vivo profile of different phase I and phase II metabolites after oral intake of an extract from *Passiflora incarnata*. Therefore, this investigation will help further clinical studies to identify in vivo metabolites and, thus, compounds responsible for the efficacy of medicinal plant extracts from plants such as *Passiflora* or *Crataegus*.

## 4. Material and Methods

### 4.1. Reagents and Chemicals

Orientin, isoorientin, vitexin, isovitexin, schaftoside, isoschaftoside, luteolin, apigenin, and quercetin (>95%, reference substances) were purchased from Phytolab (Vestenbergsgreuth, Germany). Caco-2 cells were obtained from the ATCC (American Type Culture Collection, Manassas, VA, USA). Acetonitrile (LiChrosolv^®^), trifluoroacetic acid (TFA), dimethyl sulfoxide (DMSO), formic acid, sodium acetate, sulfatase (from *Helix pomatia*, type H-1, lyophilized, >10,000 U/g), *β*-glucuronidase (from bovine liver, type B-10, 10,100 U/g) methanol (LiChrosolv^®^), 3-(4,5-dimethylthiazolyl)-2,5-diphenyl tetrazolium bromide (MTT), and sodium dodecyl sulfate (SDS) were purchased from Merck (Darmstadt, Germany). Hanks’ balanced salt solution (HBSS) was obtained from Biowest (Nuaillé, France). Dulbecco’s modified Eagle medium (DMEM) was purchased from Life Technologies (Carlsbad, CA, USA). Phosphate-buffered saline (PBS), fetal bovine serum (FBS), trypsin-ethylenediaminetetraacetic acid (trypsin-EDTA), nonessential amino acids (NEAAs), penicillin, and streptomycin were purchased from Biochrom AG (Berlin, Germany). Transwell^®^ plates (insert diameter 12 mm, pore size 3.0 µm, membrane growth area 1.12 cm^2^) were obtained from Corning Costar (Cambridge, MA, USA), and 96-well plates were purchased from TPP (Trasadingen, Switzerland).

### 4.2. Stability Testing

To ensure stability during the Caco-2 metabolism experiments, all test substances were dissolved in DMSO to a concentration of 20 mM and diluted to 10 µM in an appropriate volume of HBSS (pH 6.0), resulting in a DMSO concentration of less than 1%. The test solution (1 mL) was aliquoted into a 12-well Transwell^®^ plate and incubated for 5 h in an atmosphere of 5% CO_2_ and 90% relative humidity at 37 °C. Test samples (100 µL) were collected after t = 0, 1, 2, 3, 4, and 5 h and measured via HPLC-DAD. The peak area of the test substance at time t = 0 h was defined as 100%, and all other peak areas were related to calculate the proportional stability. The stability was calculated as % = area/area_0_ × 100. Stability testing was performed on three independent days with freshly prepared solutions (*n* = 3).

### 4.3. HPLC-DAD

The system consisted of the following: Elite LaChrom with an L2200 autosampler, L2130 pump, L2350 column oven, L2444 DAD, and EZChrom Elite 3.1.7 software (Hitachi, Tokyo, Japan); column: Kinetex^®^ Biphenyl, 100 Å, 250-4.6 mm (5 µm, same material precolumn; Phenomenex, Torrance, CA, USA); injection volume 30 µL; oven temp.: 25 °C; autosampler temp.: 10 °C; detection wavelength: 340 nm; flow: 1.2 mL/min; A = H_2_O + 0.1% TFA, B = acetonitrile + 0.1% TFA; gradient: 0–2 min 10% B, 2–10 min 10% B → 100% B, 10–11 min 100% B → 10% B, 11–15 min 10% B.

### 4.4. MTT Assay under Metabolic Conditions

Caco-2 cells in the logarithmic growth phase were seeded in 96-well plates at a density of 3 × 10^4^ cells/well and incubated at 37 °C in an atmosphere of 5% CO_2_ and constant moisture in an incubator. After 24 h, the cell culture medium was removed, and 100 µL of HBSS solution (negative control), 0.5% DMSO in HBSS solution (solvent control), or flavonoid samples in HBSS solution (6 flavonoids at concentrations of 10, 25, 50, 75, and 100 µM) was applied in quintuplicate and incubated for 5 h. Afterwards, the HBSS solutions were removed and replaced with an MTT solution in DMEM (0.4 mg/mL) and incubated for another 3 h at 37 °C. Then, the MTT solution was discarded, and the cells were fixed with a 10% SDS solution in PBS and stored at room temperature in the dark. After 24 h, the absorbance was measured on a Tecan microplate reader (Tecan Trading AG, Switzerland) at 560 nm. The viability rate was calculated as % = A/A_0_ × 100, with A as the average absorbance value of the treatment and solvent control groups and A_0_ as the average absorbance value of the negative control group. The assay was performed three times in pentaplicate.

### 4.5. Cell Culture

The human colon adenocarcinoma cell line Caco-2 was obtained from the ATCC (American Type Culture Collection, Manassas, VA, USA). The cell line was determined to be free of mycoplasma contamination by PCR and culture from GATC Biotech AG (Konstanz, Germany). The cells were cultured in DMEM supplemented with 10% (*v*/*v*) FBS, 1% (*v*/*v*) NEAA, and 1% (*v*/*v*) antibiotics (penicillin/streptomycin) in an atmosphere of 5% CO_2_ and 90% relative humidity at 37 °C. The culture medium was replaced every 3 days, and the cells were split 1:5 using trypsin-EDTA once 90% confluence was obtained.

### 4.6. Cell Differentiation

To form a confluent and differentiated monolayer, cells at passages 16–45 were seeded into the inserts of 12-well Transwell^®^ plates at a density of 3.0 × 10^5^ cells/cm^2^. Afterwards, the culture medium was replaced every day, and the metabolism experiment was performed 21 days after seeding. The apical and basolateral compartments contained 0.5 and 1.5 mL of cell culture medium, respectively.

### 4.7. Caco-2 Metabolism Experiment

On day 21, the cell culture medium was removed from the apical and basolateral compartments, and the Caco-2 cell monolayer was washed two times with prewarmed HBSS. The Transwell^®^ plates containing HBSS were incubated at 37 °C for 15 min to measure the transepithelial electrical resistance (TEER) value. The integrity of the cell monolayers was evaluated by TEER values between the apical and basolateral sides with the Evohm2 system (World Precision Instruments, Sarasota, FL, USA). The cell inserts were only used for the metabolism experiments if the resistance exceeded 350 Ω/cm^2^. The six test substances were dissolved in DMSO to a stock solution of 20 mM. The HBSS was adjusted to pH 6.0 and 7.4 with 1 M HCl. Afterwards, the stock solutions were diluted to 10 or 100 µM in an appropriate volume of HBSS (pH 6.0) to obtain a DMSO concentration of <1%. The test solutions were added to the apical side (0.5 mL), while the basolateral side was filled with 1.5 mL of HBSS (pH 7.4). The Transwell^®^ plates were incubated at 37 °C for 3 or 5 h, respectively. After this period, solutions from the apical and basolateral compartments were collected in separate tubes, washed twice with 500 µL of HBSS, and then collected again. The samples were lyophilized and stored at −20 °C until they were measured using UHPLC-DAD-MS. After the experiment, Transwell^®^ inserts were apically (0.5 mL) and basolaterally (1.5 mL) filled with HBSS and warmed to 37 °C for 20 min. Then, the TEER values were measured to guarantee the integrity of the monolayer during the experiment. The experiment was performed in triplicate for each substance, concentration, and incubation time.

### 4.8. Cell Lysis

After the metabolism experiment, cell monolayers were washed twice with PBS and incubated with 200 µL of trypsin-EDTA for 10 min at 37 °C in an atmosphere of 5% CO_2_ and constant humidity in the incubator. The cells were removed from the well by pipetting up and down, and were collected in a tube. The well was washed twice with PBS (1 mL), and the wash solution was also collected in the corresponding tube. The mixture was centrifuged (1000× *g* rpm, 5 min), and the supernatant was discarded. The pellet was washed with 1 mL of PBS and centrifuged again (1000× *g* rpm, 5 min), and the supernatant was discarded. The remaining pellet was dissolved in 400 µL of MeOH 80% (*v*/*v*), and the cells were lysed for 10 min in an ultrasonic bath. Afterwards, the solution was centrifuged (14,000× *g* rpm, 5 min), and the supernatant was dried under nitrogen. The residue was resuspended in the starting conditions, filtered (0.2 µm), and analyzed via UHPLC-DAD-MS. The experiment was performed in triplicate for each substance, concentration, and incubation time.

### 4.9. Enzymatic Sample Preparation

To cleave the conjugated phase II metabolites and identify the corresponding aglycones or C-glycosides, 1.2 mg of sulfatase and 150 µL of a 2 M sodium acetate buffer, adjusted to pH 5.0 with acetic acid and containing 10 mg/mL *β*-glucuronidase, were added to 100 µL of the metabolism samples (final concentrations of 35 U sulfatase and 3050 U *β*-glucuronidase) and filled to 1 mL with sodium acetate–acetic acid buffer. After 3 h of incubation at 37 °C, 1 mL of ice-cold ethanol was added, and the samples were vortexed for 5 min and centrifuged (14,000× *g* rpm, 5 min). The supernatant was removed and dried under nitrogen, whereas the residue was resuspended in the starting conditions, filtered, and analyzed via UHPLC-DAD-MS. The experiment was repeated three times with different samples.

### 4.10. UHPLC-DAD-MS

The UHPLC setup consisted of the following: Agilent G4220A binary pump, G4226A HiP sampler, G1316C column comp, and G4212A DAD (Agilent Technologies, Santa Clara, CA, USA); column: YMC Triart C18, 1.9 u, 75 × 2 mm, 12 nm (YMC, Kyoto, Japan); injection volume: 1 µL; oven temp.: 50 °C; autosampler temp.: 25 °C; detection wavelength: 190–640 nm; flow: 0.6 mL/min; A = H_2_O + 0.1% formic acid (FA), B = acetonitrile + 0.1% FA; and gradient: 0–10 min 8% B ➐ 15% B, 10–16 min 15% B ➐ 60% B, 16–17 min 60% B ➐ 98% B, 17–18 min 98% B, 18–18.1 min 98% B ➐ 8% B, 18.1–19.2 min 8% B. All samples were filtered (Phenex RC Membrane 0.2 µm, Phenomenex, USA) before injection.

The MS set up consisted of the following: Agilent MS Q-TOF 6540 UHD, ion source: AJS ESI (Agilent Technologies, Santa Clara, CA, USA); detection range: 80–1200 *m*/*z*; ion polarity: negative; scan rate: 3.00 spectra/s; gas temperature: 300 °C; gas flow: 8 L/min; nebulizer: 40 psi; sheath gas temperature: 300 °C; and sheath gas flow: 10 L/min. For MS/MS experiments, the collision gas was nitrogen, and the collision energy was 10, 20, or 40 eV.

All MS data were evaluated via MassHunter Qualitative Analysis B.08.00 (Agilent Technologies, Santa Clara, CA, USA). The masses of the resulting signals were compared with the exact masses of possible phase I and II metabolites collected in six compound libraries. A table of all metabolites (comprising the exact masses) and EIC (extracted ion chromatogram) peak area/time curves of detected metabolites is provided in the Supplementary Materials. The MS chromatogram peak filter parameters were as follows: ≥100 counts, ≤10 ppm, molecular feature extraction *m*/*z* range = 60–1200, ion polarity = negative, and allowed ions = (M − H)^−^.

### 4.11. Aglycone Determination

To identify the aglycones of the detected sulfated and glucuronidated conjugates, and to eliminate the failure of mass equality, the metabolites were cleaved by glucuronidase or sulfatase and analyzed by UHPLC-DAD-MS, and the retention times were compared to the corresponding reference compounds. The sulfated orientin and isoorientin monoconjugates could be confirmed through HPLC-DAD-MS/MS analysis (Tables S7–S14) and sulfatase cleavage. As an aglycone of the hydroxy-aglycone glucuronides of orientin and isoorientin, quercetin could be determined through sulfatase cleavage and comparison of the retention time (Table 1). Luteolin was identified to be the aglycone of the hydroxy-aglycone sulfate of isovitexin and of the glucuronidated monoconjugate of the C–C-cleaved isoorientin.

### 4.12. Statistical Analysis

The results are presented as the mean ± standard deviation (SD), with the mean being the average of the last three replicates. Significance levels were calculated by one-way ANOVA followed by Tukey’s HSD test using SPSS 25 (IBM, Armonk, NY, USA).

## Figures and Tables

**Table 1:** Identification of phase II metabolites using UHPLC-DAD-MS/MS fragmentation and comparison of the retention times (RTs) with a reference compound (r.c.) after enzymatic hydrolysis (a.e.h.); n.d.: not detected; n.e.: not examined; exp.: experimental.

RT (min)	(M − H)^−^ exp. (*m*/*z*)	Fragment Ions (*m*/*z*)	RT (min) Aglycone a.e.h.	RT (min) Aglycone r.c.	Possible Metabolite	Initial Substance
5.134	477.0674	301.0300 (-GlucA), 176.0107 (-Quercetin)	12.402	12.342	Quercetin-glucuronide	O, IO
5.476	461.0724	n.d.	12.437	12.425	Luteolin-glucuronide	O, IO
6.204	299.0550	285.0325 (-CH_2_)	n.e.	n.e.	Methoxy-luteolin	IV
11.658	364.9978	285.0403 (-SO_3_)	12.437	12.425	Luteolin-sulfate	IV
11.909	379.0135	299.0566 (-SO_3_), 285.0350 (-CH_2_, -SO_3_)	n.e.	n.e.	Methoxy-luteolin-sulfate	IV
3.146	527.0499	327,0485 (-C_6_H_12_O_6_, -SO_3_)	5.754	5.758	O-sulfate	O
2.514 5.107	527.0501	327.0485 (--C_6_H_12_O_6_, -SO_3_), 79.9563 (-IO)	5.926	5.947	IO-sulfate	IO
5.739 6.503	541.06574	461.1075 (-SO_3_)	n.e.	n.e.	Methoxy-IO-sulfate	IO

## Data Availability

Datasets used and/or analyzed in this study are available from the corresponding author on reasonable request.

## Supplementary Materials

The following are available online at https://www.mdpi.com/article/10.3390/ijms22126566/s1.

## Abbreviations

CYP	cytochrome P450
COMT	catechol-O-methyltransferase
SULT	sulfotransferase
UGT	uridine 5′-diphospho glucuronosyltransferase
O	orientin
IO	isoorientin
S	schaftoside
IS	isoschaftoside
V	vitexin
IV	isovitexin
DMSO	dimethyl sulfoxide
MTT	3-(4,5-dimethylthiazolyl)-2,5-diphenyl tetrazolium bromide
SDS	sodium dodecyl sulfate
HBSS	Hanks’ balanced salt solution
DMEM	Dulbecco’s modified Eagle medium
PBS	phosphate-buffered saline
FBS	fetal bovine serum
EDTA	ethylenediaminetetraacetic acid
NEAA	nonessential amino acids
TEER	transepithelial electrical resistance
(U)HPLC	(ultra) high-performance liquid chromatography
DAD	diode array detector
MS	mass spectrometry
EIC	extracted ion chromatogram
