# Supplementary material for: In Vitro Metabolism of Six C-Glycosidic Flavonoids from Passiflora incarnata L
Source: Int J Mol Sci. 2021 Jun 18;22(12):6566. doi: 10.3390/ijms22126566 (PMC8234803; doi:10.3390/ijms22126566)
Supplement: Supplementary file 1 [file ijms-22-06566-s001.zip › ijms-1258623-supplementary.pdf]

Supporting Information

In vitro metabolization of six C-glycosidic flavonoids from *Passiflora incarnata* L.

Martina Tremmel[1], Josef Kiermaier[2], Jörg Heilmann[1]

[1] University of Regensburg, Institute of Chemistry and Pharmacy, Department of Pharmaceutical Biology, Universitätsstr. 31, 93053 Regensburg, Germany

[2] University of Regensburg, Institute of Chemistry and Pharmacy, Department of Central Analytics, Universitätsstr. 31, 93053 Regensburg, Germany

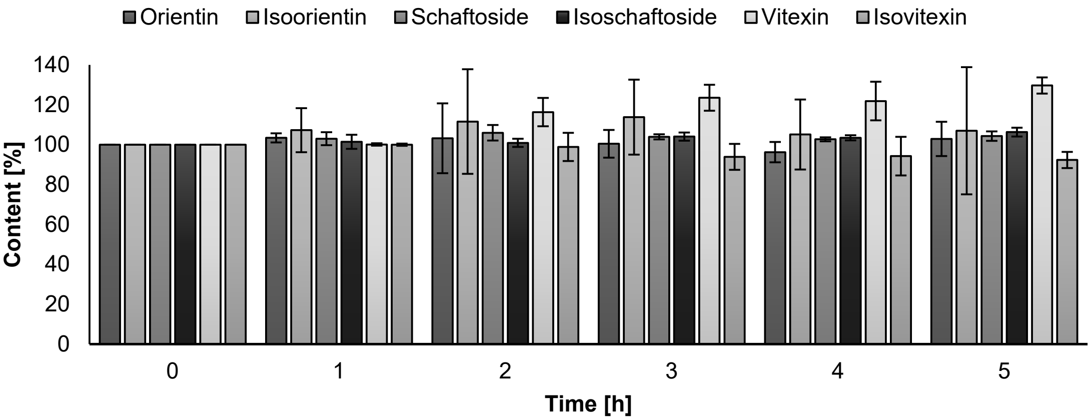

Figure S1. Stability testing of O, IO, S, IS, V and IV diluted in HBSS (pH 6.0; 10 µM) in an atmosphere of 5% CO_2 and 90% of relative humidity at 37 °C (n = 3). Data are presented as mean +/- SD.

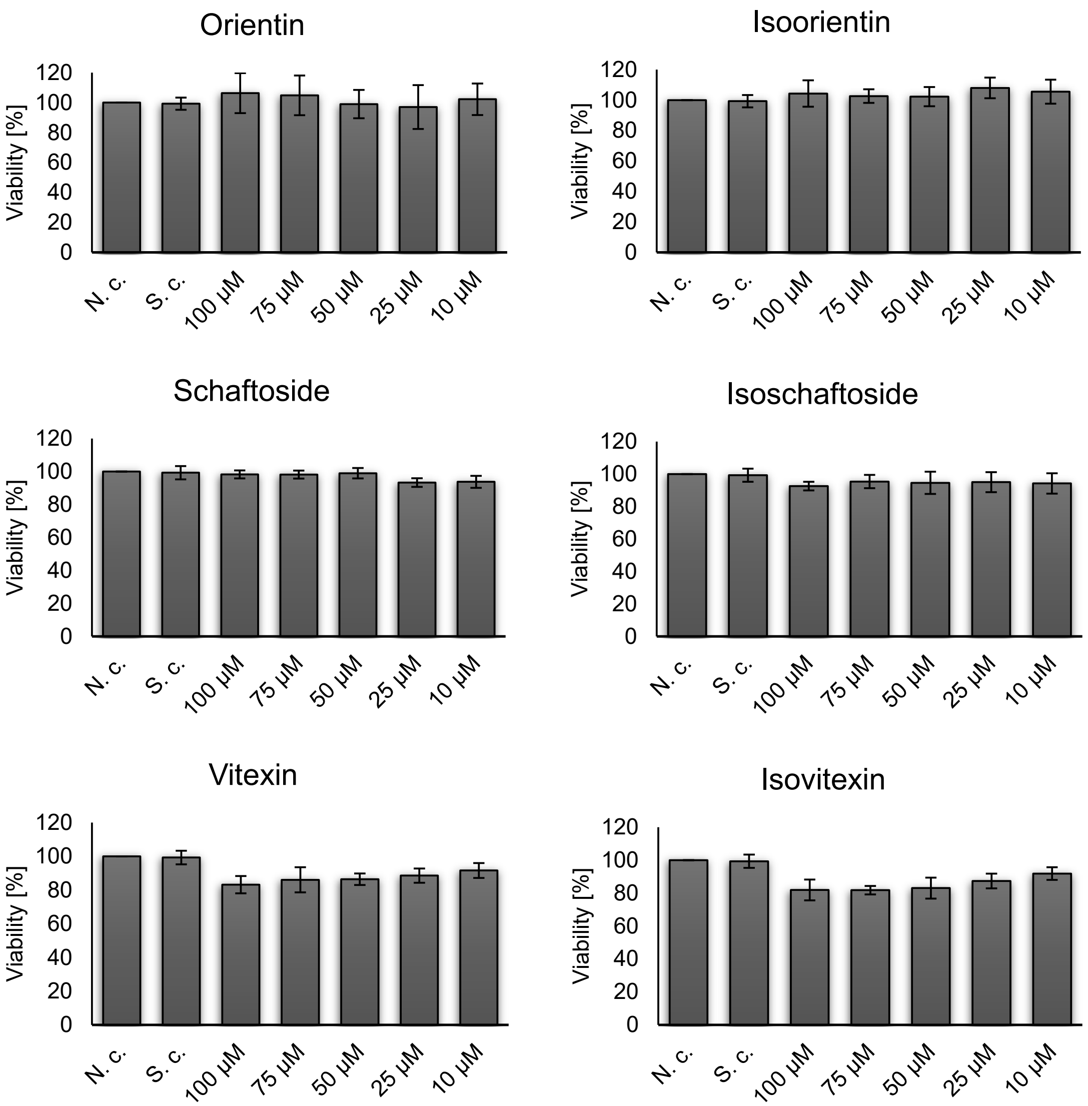

Figure S2. Results of the MTT assay under metabolization conditions. N.c.: negative control (HBSS); S.c.: solvent control (0.5% DMSO in HBSS); n = 3. Data are presented as mean +/- SD.

Table S1.: Compound library of orientin.

Molecular formula	Exact mass	Compound
$C_{21}H_{20}O_{11}$	448.1006	Orientin
$C_{15}H_{10}O_6$	286.0477	Luteolin
$C_7H_8O_2$	124.0524	Dihydroxy-toluene
$C_7H_6O_3$	138.0317	Hydroxy-benzoic acid
$C_8H_8O_3$	152.0473	Hydroxy-phenylacetic acid
$C_8H_8O_4$	168.0423	Dihydroxy-phenylacetic acid
$C_9H_{10}O_4$	182.0579	Hydroxy-methoxy-phenylacetic acid
$C_6H_6O_3$	126.0317	Phloroglucinol
$C_6H_6O_6S$	205.9885	Phloroglucinol-sulfate
$C_{12}H_{14}O_9$	302.0638	Phloroglucinol-glucuronide
$C_7H_8O_3$	140.0473	Methyl-phloroglucinol
$C_8H_8O_6S$	232.0042	Hydroxy-phenylacetic acid-sulfate
$C_{14}H_{16}O_9$	328.0794	Hydroxy-phenylacetic acid-glucuronide
$C_{10}H_{11}NO_4$	209.0688	Hydroxy-phenylacetic acid-glycine conjugate
$C_{11}H_{11}NO_4$	223.0845	Hydroxy-phenylpropionic acid-glycine conjugate
$C_9H_{10}O_6S$	246.0198	Hydroxy-phenylpropionic acid-sulfate
$C_{15}H_{18}O_9$	342.0951	Hydroxy-phenylpropionic acid-glucuronide
$C_{15}H_{16}O_9$	340.0794	Hydroxy-cinnamic acid-glucuronide
$C_9H_8O_6S$	244.0042	Hydroxy-cinnamic acid-sulfate
$C_9H_8O_4$	180.0423	Caffeic acid
$C_9H_8O_7S$	259.9991	Caffeic acid-sulfate
$C_{15}H_{16}O_{10}$	356.0743	Caffeic acid-glucuronide
$C_{10}H_{10}O_4$	194.0579	Ferulic acid
$C_{10}H_{10}O_7S$	274.0147	Ferulic acid-sulfate
$C_{16}H_{18}O_{10}$	370.0900	Ferulic acid-glucuronide
$C_9H_{10}O_4$	182.0597	Dihydroxy-phenylpropionic acid
$C_9H_{10}O_3$	166.0630	Hydroxy-phenylpropionic acid
$C_9H_8O_3$	164.0473	Hydroxy-phenylacrylic acid
$C_{10}H_{12}O_4$	196.0736	Hydroxy-methoxy-phenylpropionic acid
$C_{16}H_{12}O_6$	300.0634	Methoxy-luteolin
$C_{15}H_{10}O_7$	302.0427	Hydroxy-luteolin
$C_{16}H_{12}O_7$	316.0583	Hydroxy-methoxy-luteolin
$C_{17}H_{14}O_6$	314.0790	Dimethoxy-luteolin
$C_{18}H_{16}O_6$	328.0947	Trimethoxy-luteolin
$C_{17}H_{14}O_7$	330.0740	Hydroxy-dimethoxy-luteolin
$C_{18}H_{16}O_7$	344.0896	Hydroxy-trimethoxy-luteolin
$C_{21}H_{18}O_{12}$	462.0798	Luteolin-glucuronide
$C_{15}H_{10}O_9S$	366.0046	Luteolin-sulfate
$C_{27}H_{26}O_{18}$	638.1119	Luteolin-diglucuronide
$C_{15}H_{10}O_{12}S_2$	445.9614	Luteolin-disulfate
$C_{21}H_{18}O_5S$	542.0366	Luteolin-glucuronide-sulfate
$C_{15}H_{10}O_{10}S$	381.9995	Hydroxy-luteolin-sulfate
$C_{21}H_{18}O_{13}$	478.0747	Hydroxy-luteolin-glucuronide
$C_{22}H_{20}O_{13}$	492.0904	Hydroxy-methoxy-luteolin-glucuronide

$C_{27}H_{26}O_{19}$	654.1068	Hydroxy-luteolin-diglucuronide
$C_{21}H_{10}O_{13}S_2$	461.9563	Hydroxy-luteolin-disulfate
$C_{21}H_{18}O_{16}S$	558.0316	Hydroxy-luteolin-glucuronide-sulfate
$C_{16}H_{12}O_{10}S$	396.0151	Hydroxy-methoxy-luteolin-sulfate
$C_{16}H_{12}O_9S$	380.0202	Methoxy-luteolin-sulfate
$C_{22}H_{20}O_{12}$	476.1955	Methoxy-luteolin-glucuronide
$C_{21}H_{20}O_{12}$	464.0955	Hydroxy-orientin
$C_{22}H_{22}O_{12}$	478.1111	Hydroxy-methoxy-orientin
$C_{22}H_{22}O_{11}$	462.1162	Methoxy-orientin
$C_{21}H_{20}O_{14}S$	528.0574	Orientin-sulfate
$C_{21}H_{20}O_{15}S$	544.0523	Hydroxy-orientin-sulfate
$C_{27}H_{28}O_{18}$	640.1276	Hydroxy-orientin-glucuronide
$C_{27}H_{28}O_{17}$	624.1326	Orientin-glucuronide
$C_{22}H_{22}O_{14}S$	542.0730	Methoxy-orientin-sulfate
$C_{28}H_{30}O_{17}$	638.1483	Methoxy-orientin-glucuronide
$C_{28}H_{30}O_{18}$	654.1432	Hydroxy-methoxy-orientin-glucuronide
$C_{22}H_{22}O_{15}S$	558.0679	Hydroxy-methoxy-orientin-sulfate
$C_{27}H_{28}O_{21}S$	720.0844	Hydroxy-orientin-glucuronide-sulfate
$C_{27}H_{28}O_{20}S$	704.0895	Orientin-glucuronide-sulfate
$C_{33}H_{36}O_{23}$	800.1647	Orientin-diglucuronide
$C_{21}H_{20}O_{17}S_2$	608.0142	Orientin-disulfate
$C_{33}H_{36}O_{24}$	816.1597	Hydroxy-orientin-diglucuronide
$C_{21}H_{20}O_{18}S_2$	624.0091	Hydroxy-orientin-disulfate

Table S2.: Compound library of isoorientin.

Molecular formula	Exact mass	Compound
$C_{21}H_{20}O_{11}$	448.1006	Isoorientin
$C_{15}H_{10}O_{6}$	286.0477	Luteolin
$C_{7}H_{8}O_{2}$	124.0524	Dihydroxy-toluene
$C_{7}H_{6}O_{3}$	138.0317	Hydroxy-benzoic acid
$C_{8}H_{8}O_{3}$	152.0473	Hydroxy-phenylacetic acid
$C_{8}H_{8}O_{4}$	168.0423	Dihydroxy-phenylacetic acid
$C_{9}H_{10}O_{4}$	182.0579	Hydroxy-methoxy-phenylacetic acid
$C_{6}H_{6}O_{3}$	126.0317	Phloroglucinol
$C_{6}H_{6}O_{6}S$	205.9885	Phloroglucinol-sulfate
$C_{12}H_{14}O_{9}$	302.0638	Phloroglucinol-glucuronide
$C_{7}H_{8}O_{3}$	140.0473	Methyl-phloroglucinol
$C_{8}H_{8}O_{6}S$	232.0042	Hydroxy-phenylacetic acid-sulfate
$C_{14}H_{16}O_{9}$	328.0794	Hydroxy-phenylacetic acid-glucuronide
$C_{10}H_{11}NO_{4}$	209.0688	Hydroxy-phenylacetic acid-glycine conjugate
$C_{11}H_{11}NO_{4}$	223.0845	Hydroxy-phenylpropionic acid-glycine conjugate
$C_{9}H_{10}O_{6}S$	246.0198	Hydroxy-phenylpropionic acid-sulfate
$C_{15}H_{18}O_{9}$	342.0951	Hydroxy-phenylpropionic acid-glucuronide
$C_{15}H_{16}O_{9}$	340.0794	Hydroxy-cinnamic acid-glucuronide
$C_{9}H_{8}O_{6}S$	244.0042	Hydroxy-cinnamic acid-sulfate
$C_{9}H_{8}O_{4}$	180.0423	Caffeic acid
$C_{9}H_{8}O_{7}S$	259.9991	Caffeic acid-sulfate
$C_{15}H_{16}O_{10}$	356.0743	Caffeic acid-glucuronide
$C_{10}H_{10}O_{4}$	194.0579	Ferulic acid
$C_{10}H_{10}O_{7}S$	274.0147	Ferulic acid-sulfate
$C_{16}H_{18}O_{10}$	370.0900	Ferulic acid-glucuronide
$C_{9}H_{10}O_{4}$	182.0597	Dihydroxy-phenylpropionic acid
$C_{9}H_{10}O_{3}$	166.0630	Hydroxy-phenylpropionic acid
$C_{9}H_{8}O_{3}$	164.0473	Hydroxy-phenylacrylic acid
$C_{10}H_{12}O_{4}$	196.0736	Hydroxy-methoxy-phenylpropionic acid
$C_{16}H_{12}O_{6}$	300.0634	Methoxy-luteolin
$C_{15}H_{10}O_{7}$	302.0427	Hydroxy-luteolin
$C_{16}H_{12}O_{7}$	316.0583	Hydroxy-methoxy-luteolin
$C_{17}H_{14}O_{6}$	314.0790	Dimethoxy-luteolin
$C_{18}H_{16}O_{6}$	328.0947	Trimethoxy-luteolin
$C_{17}H_{14}O_{7}$	330.0740	Hydroxy-dimethoxy-luteolin
$C_{18}H_{16}O_{7}$	344.0896	Hydroxy-trimethoxy-luteolin
$C_{21}H_{18}O_{12}$	462.0798	Luteolin-glucuronide
$C_{15}H_{10}O_{9}S$	366.0046	Luteolin-sulfate
$C_{27}H_{26}O_{18}$	638.1119	Luteolin-diglucuronide
$C_{15}H_{10}O_{12}S_{2}$	445.9614	Luteolin-disulfate
$C_{21}H_{18}O_{5}S$	542.0366	Luteolin-glucuronide-sulfate
$C_{15}H_{10}O_{10}S$	381.9995	Hydroxy-luteolin-sulfate
$C_{21}H_{18}O_{13}$	478.0747	Hydroxy-luteolin-glucuronide
$C_{22}H_{20}O_{13}$	492.0904	Hydroxy-methoxy-luteolin-glucuronide

$C_{27}H_{26}O_{19}$	654.1068	Hydroxy-luteolin-diglucuronide
$C_{21}H_{10}O_{13}S_2$	461.9563	Hydroxy-luteolin-disulfate
$C_{21}H_{18}O_{16}S$	558.0316	Hydroxy-luteolin-glucuronide-sulfate
$C_{16}H_{12}O_{10}S$	396.0151	Hydroxy-methoxy-luteolin-sulfate
$C_{16}H_{12}O_9S$	380.0202	Methoxy-luteolin-sulfate
$C_{22}H_{20}O_{12}$	476.1955	Methoxy-luteolin-glucuronide
$C_{21}H_{20}O_{12}$	464.0955	Hydroxy-orientin
$C_{22}H_{22}O_{12}$	478.1111	Hydroxy-methoxy-orientin
$C_{22}H_{22}O_{11}$	462.1162	Methoxy-orientin
$C_{21}H_{20}O_{14}S$	528.0574	Orientin-sulfate
$C_{21}H_{20}O_{15}S$	544.0523	Hydroxy-isoorientin-sulfate
$C_{27}H_{28}O_{18}$	640.1276	Hydroxy-isoorientin-glucuronide
$C_{27}H_{28}O_{17}$	624.1326	Isoorientin-glucuronide
$C_{22}H_{22}O_{14}S$	542.0730	Methoxy-isoorientin-sulfate
$C_{28}H_{30}O_{17}$	638.1483	Methoxy-isoorientin-glucuronide
$C_{28}H_{30}O_{18}$	654.1432	Hydroxy-methoxy-isoorientin-glucuronide
$C_{22}H_{22}O_{15}S$	558.0679	Hydroxy-methoxy-isoorientin-sulfate
$C_{27}H_{28}O_{21}S$	720.0844	Hydroxy-isoorientin-glucuronide-sulfate
$C_{27}H_{28}O_{20}S$	704.0895	Isoorientin-glucuronide-sulfate
$C_{33}H_{36}O_{23}$	800.1647	Isoorientin-diglucuronide
$C_{21}H_{20}O_{17}S_2$	608.0142	Isoorientin-disulfate
$C_{33}H_{36}O_{24}$	816.1597	Hydroxy-isoorientin-diglucuronide
$C_{21}H_{20}O_{18}S_2$	624.0091	Hydroxy-isoorientin-disulfate

Table S3.: Compound library of schaftoside.

Molecular formula	Exact mass	Compound
$C_{26}H_{28}O_{14}$	564.1479	Schaftoside
$C_{15}H_{10}O_5$	270.528	Apigenin
$C_7H_8O_2$	124.0524	Dihydroxy-toluene
$C_7H_6O_3$	138.0317	Hydroxy-benzoic acid
$C_8H_8O_3$	152.0473	Hydroxy-phenylacetic acid
$C_8H_8O_4$	168.0423	Dihydroxy-phenylacetic acid
$C_9H_{10}O_4$	182.0579	Hydroxy-methoxy-phenylacetic acid
$C_6H_6O_3$	126.0317	Phloroglucinol
$C_6H_6O_6S$	205.9885	Phloroglucinol-sulfate
$C_{12}H_{14}O_9$	302.0638	Phloroglucinol-glucuronide
$C_7H_8O_3$	140.0473	Methyl-phloroglucinol
$C_8H_8O_6S$	232.0042	Hydroxy-phenylacetic acid-sulfate
$C_{14}H_{16}O_9$	328.0794	Hydroxy-phenylacetic acid-glucuronide
$C_{10}H_{11}NO_4$	209.0688	Hydroxy-phenylacetic acid-glycine conjugate
$C_{11}H_{11}NO_4$	223.0845	Hydroxy-phenylpropionic acid-glycine conjugate
$C_9H_{10}O_6S$	246.0198	Hydroxy-phenylpropionic acid-sulfate
$C_{15}H_{18}O_9$	342.0951	Hydroxy-phenylpropionic acid-glucuronide
$C_{15}H_{16}O_9$	340.0794	Hydroxy-cinnamic acid-glucuronide
$C_9H_8O_6S$	244.0042	Hydroxy-cinnamic acid-sulfate
$C_9H_8O_4$	180.0423	Caffeic acid
$C_9H_8O_7S$	259.9991	Caffeic acid-sulfate
$C_{15}H_{16}O_{10}$	356.0743	Caffeic acid-glucuronide
$C_{10}H_{10}O_4$	194.0579	Ferulic acid
$C_{10}H_{10}O_7S$	274.0147	Ferulic acid-sulfate
$C_{16}H_{18}O_{10}$	370.0900	Ferulic acid-glucuronide
$C_9H_{10}O_4$	182.0597	Dihydroxy-phenylpropionic acid
$C_9H_{10}O_3$	166.0630	Hydroxy-phenylpropionic acid
$C_9H_8O_3$	164.0473	Hydroxy-phenylacrylic acid
$C_{10}H_{12}O_4$	196.0736	Hydroxy-methoxy-phenylpropionic acid
$C_{16}H_{12}O_5$	284.0685	Methoxy-apigenin
$C_{17}H_{14}O_5$	298.0841	Dimethoxy-apigenin
$C_{15}H_{10}O_6$	286.477	Hydroxy-apigenin
$C_{16}H_{12}O_6$	300.0634	Hydroxy-methoxy-apigenin
$C_{17}H_{14}O_6$	314.0790	Hydroxy-dimethoxy-apigeninr
$C_{18}H_{16}O_6$	328.0947	Hydroxy-trimethoxy-apigeninr
$C_{15}H_{10}O_8S$	350.0096	Apigenin-sulfate
$C_{21}H_{18}O_{11}$	446.0849	Apigenin-glucuronide
$C_{27}H_{26}O_{17}$	622.1170	Apigenin-diglucuronide
$C_{15}H_{10}O_{11}S_2$	429.9665	Apigenin-disulfate
$C_{21}H_{18}O_{14}S$	526.0417	Apigenin-glucuronide-sulfate
$C_{15}H_{10}O_9S$	366.0046	Hydroxy-apigenin-sulfate
$C_{16}H_{12}O_8S$	364.0253	Methoxy-apigenin-sulfate
$C_{16}H_{12}O_9S$	380.0202	Hydroxy-methoxy-apigenin-sulfate
$C_{22}H_{20}O_{11}$	460.1006	Methoxy-apigenin-glucuronide

$C_{21}H_{18}O_{12}$	462.0798	Hydroxy-apigenin-glucuronide
$C_{22}H_{20}O_{12}$	476.0955	Hydroxy-methoxy-apigenin-glucuronide
$C_{15}H_{10}O_{12}S_2$	445.9614	Hydroxy-apigenin-disulfate
$C_{27}H_{28}O_{18}$	638.1119	Hydroxy-apigenin-diglucuronide
$C_{21}H_{18}O_{15}S$	542.0366	Hydroxy-apigenin-glucuronide-sulfate
$C_{27}H_{30}O_{14}$	578.1636	Methoxy-schaftoside
$C_{27}H_{30}O_{15}$	594.1585	Hydroxy-methoxy-schaftoside
$C_{26}H_{28}O_{15}$	580.1428	Hydroxy-schaftoside
$C_{28}H_{32}O_{14}$	592.1792	Dimethoxy-schaftoside
$C_{28}H_{32}O_{15}$	608.1741	Hydroxy-dimethoxy-schaftoside
$C_{26}H_{28}O_{17}S$	644.1047	Schaftoside-sulfate
$C_{26}H_{28}O_{20}S_2$	724.0618	Schaftoside-disulfate
$C_{26}H_{28}O_{21}S_2$	740.0564	Hydroxy-schaftoside-disulfate
$C_{32}H_{36}O_{20}$	740.1800	Schaftoside-glucuronide
$C_{32}H_{26}O_{23}S$	820.1368	Schaftoside-glucuronide-sulfate
$C_{38}H_{44}O_{26}$	916.2121	Schaftoside-diglucuronide
$C_{32}H_{36}O_{24}S$	836.1317	Hydroxy-schaftoside-glucuronide-sulfate
$C_{38}H_{44}O_{27}$	932.2070	Hydroxy-schaftoside-diglucuronide
$C_{33}H_{38}O_{20}$	754.1956	Methoxy-schaftoside-glucuronide
$C_{32}H_{36}O_{21}$	756.1749	Hydroxy-schaftoside-glucuronide
$C_{33}H_{38}O_{21}$	770.1906	Hydroxy-methoxy-schaftoside-glucuronide
$C_{27}H_{30}O_{17}S$	658.1204	Methoxy-schaftoside-sulfate
$C_{26}H_{28}O_{18}S$	660.0996	Hydroxy-schaftoside-sulfate
$C_{27}H_{30}O_{18}S$	674.1153	Hydroxy-methoxy-schaftoside-sulfate
$C_{22}H_{22}O_{10}$	446.1213	Methoxy-apigenin-8-C-riboside
$C_{22}H_{22}O_{11}$	462.1162	Hydroxy-methoxy-apigenin-8-C-riboside
$C_{21}H_{20}O_{11}$	448.1006	Hydroxy-apigenin-8-C-riboside
$C_{23}H_{24}O_{10}$	460.1369	Dimethoxy-apigenin-8-C-riboside
$C_{23}H_{24}O_{11}$	476.1319	Hydroxy-dimethoxy-apigenin-8-C-riboside
$C_{21}H_{20}O_{13}S$	512.0625	Apigenin-8-C-riboside-sulfate
$C_{21}H_{20}O_{16}S_2$	592.0193	Apigenin-8-C-riboside-disulfate
$C_{21}H_{20}O_{17}S_2$	608.0142	Hydroxy-apigenin-8-C-riboside-disulfate
$C_{27}H_{28}O_{16}$	608.1377	Apigenin-8-C-riboside-glucuronide
$C_{27}H_{28}O_{19}S$	688.0945	Apigenin-8-C-riboside-glucuronide-sulfate
$C_{33}H_{36}O_{22}$	784.1698	Apigenin-8-C-riboside-diglucuronide
$C_{27}H_{28}O_{20}S$	704.0895	Hydroxy-apigenin-8-C-riboside-glucuronide-sulfate
$C_{33}H_{36}O_{23}$	800.1647	Hydroxy-apigenin-8-C-riboside-diglucuronide
$C_{28}H_{30}O_{16}$	622.1534	Methoxy- apigenin-8-C-riboside-glucuronide
$C_{27}H_{28}O_{17}$	624.1326	Hydroxy-apigenin-8-C-riboside-glucuronide
$C_{28}H_{30}O_{17}$	638.1483	Hydroxy-methoxy- apigenin-8-C-riboside-glucuronide
$C_{22}H_{22}O_{13}S$	526.0781	Methoxy- apigenin-8-C-riboside-sulfate
$C_{21}H_{20}O_{14}S$	528.0574	Hydroxy-apigenin-8-C-riboside-sulfate
$C_{22}H_{22}O_{14}S$	542.0730	Hydroxy-methoxy-apigenin-8-C-riboside-sulfat
$C_{21}H_{20}O_{9}$	416.1107	Methoxy-apigenin-6-C-glucoside
$C_{21}H_{20}O_{10}$	432.1056	Hydroxy-methoxy- apigenin-6-C-glucoside
$C_{20}H_{18}O_{10}$	418.0900	Hydroxy-apigenin-6-C-glucoside
$C_{22}H_{22}O_{9}$	430.1264	Dimethoxy-apigenin-6-C-glucoside

$C_{20}H_{20}O_{10}$	446.1213	Hydroxy-dimethoxy-apigenin-6-C-glucoside
$C_{20}H_{18}O_{12}S$	482.0519	Apigenin-6-C-glucoside-sulfate
$C_{20}H_{18}O_{15}S_2$	562.0087	Apigenin-6-C-glucoside-disulfate
$C_{20}H_{18}O_{16}S_2$	578.0036	Hydroxy-apigenin-6-C-glucoside-disulfate
$C_{26}H_{26}O_{15}$	578.1272	Apigenin-6-C-glucoside-glucuronide
$C_{26}H_{26}O_{18}S$	658.0840	Apigenin-6-C-glucoside-glucuronide-sulfate
$C_{33}H_{34}O_{21}$	754.1593	Apigenin-6-C-glucoside-diglucuronide
$C_{26}H_{26}O_{19}S$	674.0789	Hydroxy-apigenin-6-C-glucoside-glucuronide-sulfate
$C_{32}H_{34}O_{22}$	770.1542	Hydroxy-apigenin-6-C-glucoside-diglucuronide
$C_{27}H_{28}O_{15}$	592.1428	Methoxy- Apigenin-6-C-glucoside-glucuronide
$C_{26}H_{26}O_{16}$	594.1221	Hydroxy-apigenin-6-C-glucoside-glucuronide
$C_{27}H_{28}O_{16}$	608.1377	Hydroxy-methoxy-apigenin-6-C-glucoside-glucuronide
$C_{21}H_{20}O_{12}S$	496.0675	Methoxy-apigenin-6-C-glucoside-sulfate
$C_{20}H_{18}O_{13}S$	498.0468	Hydroxy-apigenin-6-C-glucoside-sulfate
$C_{21}H_{20}O_{13}S$	512.0625	Hydroxy-methoxy-apigenin-6-C-glucoside-sulfate

Table S4.: Compound library of isoschaftoside.

Molecular formula	Exact mass	Compound
$C_{26}H_{28}O_{14}$	564.1479	Isoschaftoside
$C_{15}H_{10}O_5$	270.528	Apigenin
$C_7H_8O_2$	124.0524	Dihydroxy-toluene
$C_7H_6O_3$	138.0317	Hydroxy-benzoic acid
$C_8H_8O_3$	152.0473	Hydroxy-phenylacetic acid
$C_8H_8O_4$	168.0423	Dihydroxy-phenylacetic acid
$C_9H_{10}O_4$	182.0579	Hydroxy-methoxy-phenylacetic acid
$C_6H_6O_3$	126.0317	Phloroglucinol
$C_6H_6O_6S$	205.9885	Phloroglucinol-sulfate
$C_{12}H_{14}O_9$	302.0638	Phloroglucinol-glucuronide
$C_7H_8O_3$	140.0473	Methyl-phloroglucinol
$C_8H_8O_6S$	232.0042	Hydroxy-phenylacetic acid-sulfate
$C_{14}H_{16}O_9$	328.0794	Hydroxy-phenylacetic acid-glucuronide
$C_{10}H_{11}NO_4$	209.0688	Hydroxy-phenylacetic acid-glycine conjugate
$C_{11}H_{11}NO_4$	223.0845	Hydroxy-phenylpropionic acid-glycine conjugate
$C_9H_{10}O_6S$	246.0198	Hydroxy-phenylpropionic acid-sulfate
$C_{15}H_{18}O_9$	342.0951	Hydroxy-phenylpropionic acid-glucuronide
$C_{15}H_{16}O_9$	340.0794	Hydroxy-cinnamic acid-glucuronide
$C_9H_8O_6S$	244.0042	Hydroxy-cinnamic acid-sulfate
$C_9H_8O_4$	180.0423	Caffeic acid
$C_9H_8O_7S$	259.9991	Caffeic acid-sulfate
$C_{15}H_{16}O_{10}$	356.0743	Caffeic acid-glucuronide
$C_{10}H_{10}O_4$	194.0579	Ferulic acid
$C_{10}H_{10}O_7S$	274.0147	Ferulic acid-sulfate
$C_{16}H_{18}O_{10}$	370.0900	Ferulic acid-glucuronide
$C_9H_{10}O_4$	182.0597	Dihydroxy-phenylpropionic acid
$C_9H_{10}O_3$	166.0630	Hydroxy-phenylpropionic acid
$C_9H_8O_3$	164.0473	Hydroxy-phenylacrylic acid
$C_{10}H_{12}O_4$	196.0736	Hydroxy-methoxy-phenylpropionic acid
$C_{16}H_{12}O_5$	284.0685	Methoxy-apigenin
$C_{17}H_{14}O_5$	298.0841	Dimethoxy-apigenin
$C_{15}H_{10}O_6$	286.4770	Hydroxy-apigenin
$C_{16}H_{12}O_6$	300.0634	Hydroxy-methoxy-apigenin
$C_{17}H_{14}O_6$	314.0790	Hydroxy-dimethoxy-apigeninr
$C_{18}H_{16}O_6$	328.0947	Hydroxy-trimethoxy-apigeninr
$C_{15}H_{10}O_8S$	350.0096	Apigenin-sulfate
$C_{21}H_{18}O_{11}$	446.0849	Apigenin-glucuronide
$C_{27}H_{26}O_{17}$	622.1170	Apigenin-diglucuronide
$C_{15}H_{10}O_{11}S_2$	429.9665	Apigenin-disulfate
$C_{21}H_{18}O_{14}S$	526.0417	Apigenin-glucuronide-sulfate
$C_{15}H_{10}O_9S$	366.0046	Hydroxy-apigenin-sulfate
$C_{16}H_{12}O_8S$	364.0253	Methoxy-apigenin-sulfate
$C_{16}H_{12}O_9S$	380.0202	Hydroxy-methoxy-apigenin-sulfate
$C_{22}H_{20}O_{11}$	460.1006	Methoxy-apigenin-glucuronide

$C_{21}H_{18}O_{12}$	462.0798	Hydroxy-apigenin-glucuronide
$C_{22}H_{20}O_{12}$	476.0955	Hydroxy-methoxy-apigenin-glucuronide
$C_{15}H_{10}O_{12}S_2$	445.9614	Hydroxy-apigenin-disulfate
$C_{27}H_{28}O_{18}$	638.1119	Hydroxy-apigenin-diglucuronide
$C_{21}H_{18}O_{15}S$	542.0366	Hydroxy-apigenin-glucuronide-sulfate
$C_{27}H_{30}O_{14}$	578.1636	Methoxy-isoschaftoside
$C_{27}H_{30}O_{15}$	594.1585	Hydroxy-methoxy-isoschaftoside
$C_{26}H_{28}O_{15}$	580.1428	Hydroxy-isoschaftoside
$C_{28}H_{32}O_{14}$	592.1792	Dimethoxy-isoschaftoside
$C_{28}H_{32}O_{15}$	608.1741	Hydroxy-dimethoxy-isoschaftoside
$C_{26}H_{28}O_{17}S$	644.1047	Isoschaftoside-sulfate
$C_{26}H_{28}O_{20}S_2$	724.0618	Isoschaftoside-disulfate
$C_{26}H_{28}O_{21}S_2$	740.0564	Hydroxy-isoschaftoside-disulfate
$C_{32}H_{36}O_{20}$	740.1800	Isoschaftoside-glucuronide
$C_{32}H_{26}O_{23}S$	820.1368	Isoschaftoside-glucuronide-sulfate
$C_{38}H_{44}O_{26}$	916.2121	Isoschaftoside-diglucuronide
$C_{32}H_{36}O_{24}S$	836.1317	Hydroxy-isoschaftoside-glucuronide-sulfate
$C_{38}H_{44}O_{27}$	932.2070	Hydroxy-isoschaftoside-diglucuronide
$C_{33}H_{38}O_{20}$	754.1956	Methoxy-isoschaftoside-glucuronide
$C_{32}H_{36}O_{21}$	756.1749	Hydroxy-isoschaftoside-glucuronide
$C_{33}H_{38}O_{21}$	770.1906	Hydroxy-methoxy-isoschaftoside-glucuronide
$C_{27}H_{30}O_{17}S$	658.1204	Methoxy-isoschaftoside-sulfate
$C_{26}H_{28}O_{18}S$	660.0996	Hydroxy-isoschaftoside-sulfate
$C_{27}H_{30}O_{18}S$	674.1153	Hydroxy-methoxy-isoschaftoside-sulfate
$C_{22}H_{22}O_{10}$	446.1213	Methoxy-apigenin-6-C-riboside
$C_{22}H_{22}O_{11}$	462.1162	Hydroxy-methoxy-apigenin-6-C-riboside
$C_{21}H_{20}O_{11}$	448.1006	Hydroxy-apigenin-6-C-riboside
$C_{23}H_{24}O_{10}$	460.1369	Dimethoxy-apigenin-6-C-riboside
$C_{23}H_{24}O_{11}$	476.1319	Hydroxy-dimethoxy-apigenin-6-C-riboside
$C_{21}H_{20}O_{13}S$	512.0625	Apigenin-6-C-riboside-sulfate
$C_{21}H_{20}O_{16}S_2$	592.0193	Apigenin-6-C-riboside-disulfate
$C_{21}H_{20}O_{17}S_2$	608.0142	Hydroxy-apigenin-6-C-riboside-disulfate
$C_{27}H_{28}O_{16}$	608.1377	Apigenin-6-C-riboside-glucuronide
$C_{27}H_{28}O_{19}S$	688.0945	Apigenin-6-C-riboside-glucuronide-sulfate
$C_{33}H_{36}O_{22}$	784.1698	Apigenin-6-C-riboside-diglucuronide
$C_{27}H_{28}O_{20}S$	704.0895	Hydroxy-apigenin-6-C-riboside-glucuronide-sulfate
$C_{33}H_{36}O_{23}$	800.1647	Hydroxy-apigenin-6-C-riboside-diglucuronide
$C_{28}H_{30}O_{16}$	622.1534	Methoxy- apigenin-6-C-riboside-glucuronide
$C_{27}H_{28}O_{17}$	624.1326	Hydroxy-apigenin-6-C-riboside-glucuronide
$C_{28}H_{30}O_{17}$	638.1483	Hydroxy-methoxy- apigenin-6-C-riboside-glucuronide
$C_{22}H_{22}O_{13}S$	526.0781	Methoxy- apigenin-6-C-riboside-sulfate
$C_{21}H_{20}O_{14}S$	528.0574	Hydroxy-apigenin-6-C-riboside-sulfate
$C_{22}H_{22}O_{14}S$	542.0730	Hydroxy-methoxy-apigenin-6-C-riboside-sulfat
$C_{21}H_{20}O_9$	416.1107	Methoxy-apigenin-8-C-glucoside
$C_{21}H_{20}O_{10}$	432.1056	Hydroxy-methoxy- apigenin-8-C-glucoside
$C_{20}H_{18}O_{10}$	418.0900	Hydroxy-apigenin-8-C-glucoside
$C_{22}H_{22}O_9$	430.1264	Dimethoxy-apigenin-8-C-glucoside

$C_{20}H_{20}O_{10}$	446.1213	Hydroxy-dimethoxy-apigenin-8-C-glucoside
$C_{20}H_{18}O_{12}S$	482.0519	Apigenin-8-C-glucoside-sulfate
$C_{20}H_{18}O_{15}S_2$	562.0087	Apigenin-8-C-glucoside-disulfate
$C_{20}H_{18}O_{16}S_2$	578.0036	Hydroxy-apigenin-8-C-glucoside-disulfate
$C_{26}H_{26}O_{15}$	578.1272	Apigenin-8-C-glucoside-glucuronide
$C_{26}H_{26}O_{18}S$	658.0840	Apigenin-8-C-glucoside-glucuronide-sulfate
$C_{33}H_{34}O_{21}$	754.1593	Apigenin-8-C-glucoside-diglucuronide
$C_{26}H_{26}O_{19}S$	674.0789	Hydroxy-apigenin-8-C-glucoside-glucuronide-sulfate
$C_{32}H_{34}O_{22}$	770.1542	Hydroxy-apigenin-8-C-glucoside-diglucuronide
$C_{27}H_{28}O_{15}$	592.1428	Methoxy- Apigenin-8-C-glucoside-glucuronide
$C_{26}H_{26}O_{16}$	594.1221	Hydroxy-apigenin-8-C-glucoside-glucuronide
$C_{27}H_{28}O_{16}$	608.1377	Hydroxy-methoxy-apigenin-8-C-glucoside-glucuronide
$C_{21}H_{20}O_{12}S$	496.0675	Methoxy-apigenin-8-C-glucoside-sulfate
$C_{20}H_{18}O_{13}S$	498.0468	Hydroxy-apigenin-8-C-glucoside-sulfate
$C_{21}H_{20}O_{13}S$	512.0625	Hydroxy-methoxy-apigenin-8-C-glucoside-sulfate

Table S5.: Compound library of vitexin.

Molecular formula	Exact mass	Compound
$C_{21}H_{20}O_{10}$	432.1056	Vitexin
$C_{15}H_{10}O_5$	270.528	Apigenin
$C_7H_8O_2$	124.0524	Dihydroxy-toluene
$C_7H_6O_3$	138.0317	Hydroxy-benzoic acid
$C_8H_8O_3$	152.0473	Hydroxy-phenylacetic acid
$C_8H_8O_4$	168.0423	Dihydroxy-phenylacetic acid
$C_9H_{10}O_4$	182.0579	Hydroxy-methoxy-phenylacetic acid
$C_6H_6O_3$	126.0317	Phloroglucinol
$C_6H_6O_6S$	205.9885	Phloroglucinol-sulfate
$C_{12}H_{14}O_9$	302.0638	Phloroglucinol-glucuronide
$C_7H_8O_3$	140.0473	Methyl-phloroglucinol
$C_8H_8O_6S$	232.0042	Hydroxy-phenylacetic acid-sulfate
$C_{14}H_{16}O_9$	328.0794	Hydroxy-phenylacetic acid-glucuronide
$C_{10}H_{11}NO_4$	209.0688	Hydroxy-phenylacetic acid-glycine conjugate
$C_{11}H_{11}NO_4$	223.0845	Hydroxy-phenylpropionic acid-glycine conjugate
$C_9H_{10}O_6S$	246.0198	Hydroxy-phenylpropionic acid-sulfate
$C_{15}H_{18}O_9$	342.0951	Hydroxy-phenylpropionic acid-glucuronide
$C_{15}H_{16}O_9$	340.0794	Hydroxy-cinnamic acid-glucuronide
$C_9H_8O_6S$	244.0042	Hydroxy-cinnamic acid-sulfate
$C_9H_8O_4$	180.0423	Caffeic acid
$C_9H_8O_7S$	259.9991	Caffeic acid-sulfate
$C_{15}H_{16}O_{10}$	356.0743	Caffeic acid-glucuronide
$C_{10}H_{10}O_4$	194.0579	Ferulic acid
$C_{10}H_{10}O_7S$	274.0147	Ferulic acid-sulfate
$C_{16}H_{18}O_{10}$	370.0900	Ferulic acid-glucuronide
$C_9H_{10}O_4$	182.0597	Dihydroxy-phenylpropionic acid
$C_9H_{10}O_3$	166.0630	Hydroxy-phenylpropionic acid
$C_9H_8O_3$	164.0473	Hydroxy-phenylacrylic acid
$C_{10}H_{12}O_4$	196.0736	Hydroxy-methoxy-phenylpropionic acid
$C_{16}H_{12}O_5$	284.0685	Methoxy-apigenin
$C_{17}H_{14}O_5$	298.0841	Dimethoxy-apigenin
$C_{15}H_{10}O_6$	286.477	Hydroxy-apigenin
$C_{16}H_{12}O_6$	300.0634	Hydroxy-methoxy-apigenin
$C_{17}H_{14}O_6$	314.0790	Hydroxy-dimethoxy-apigenin
$C_{18}H_{16}O_6$	328.0947	Hydroxy-trimethoxy-apigenin
$C_{15}H_{10}O_8S$	350.0096	Apigenin-sulfate
$C_{21}H_{18}O_{11}$	446.0849	Apigenin-glucuronide
$C_{27}H_{26}O_{17}$	622.1170	Apigenin-diglucuronide
$C_{15}H_{10}O_{11}S_2$	429.9665	Apigenin-disulfate
$C_{21}H_{18}O_{14}S$	526.0417	Apigenin-glucuronide-sulfate
$C_{15}H_{10}O_9S$	366.0046	Hydroxy-apigenin-sulfate
$C_{16}H_{12}O_8S$	364.0253	Methoxy-apigenin-sulfate
$C_{16}H_{12}O_9S$	380.0202	Hydroxy-methoxy-apigenin-sulfate
$C_{22}H_{20}O_{11}$	460.1006	Methoxy-apigenin-glucuronide

$C_{21}H_{18}O_{12}$	462.0798	Hydroxy-apigenin-glucuronide
$C_{22}H_{20}O_{12}$	476.0955	Hydroxy-methoxy-apigenin-glucuronide
$C_{15}H_{10}O_{12}S_2$	445.9614	Hydroxy-apigenin-disulfate
$C_{27}H_{28}O_{18}$	638.1119	Hydroxy-apigenin-diglucuronide
$C_{21}H_{18}O_{15}S$	542.0366	Hydroxy-apigenin-glucuronide-sulfate
$C_{22}H_{22}O_{10}$	446.1213	Methoxy-vitexin
$C_{23}H_{24}O_{10}$	460.1369	Dimethoxy-vitexin
$C_{21}H_{20}O_{11}$	448.1006	Hydroxy-vitexin
$C_{21}H_{20}O_{12}$	464.0955	Dihydroxy-vitexin
$C_{22}H_{22}O_{11}$	462.1162	Hydroxy-methoxy-vitexin
$C_{23}H_{24}O_{11}$	476.1319	Hydroxy-dimethoxy-vitexin
$C_{22}H_{22}O_{12}$	478.1111	Dihydroxy-methoxy-vitexin
$C_{23}H_{24}O_{12}$	492.1268	Dihydroxy-dimethoxy-vitexin
$C_{21}H_{20}O_{13}S$	512.0625	Vitexin-sulfate
$C_{22}H_{22}O_{13}S$	526.0781	Methoxy-vitexin-sulfate
$C_{21}H_{20}O_{14}S$	528.0574	Hydroxy-vitexin-sulfate
$C_{22}H_{20}O_{14}S$	542.0730	Hydroxy-methoxy-vitexin-sulfate
$C_{21}H_{20}O_{16}S_2$	592.0193	Vitexin-disulfate
$C_{27}H_{28}O_{16}$	608.1377	Vitexin-glucuronide
$C_{28}H_{30}O_{16}$	622.1534	Methoxy-vitexin-glucuronide
$C_{27}H_{28}O_{17}$	624.1326	Hydroxy-vitexin-glucuronide
$C_{28}H_{30}O_{17}$	638.1483	Hydroxy-methoxy-vitexin-glucuronide
$C_{33}H_{36}O_{22}$	784.1698	Vitexin-diglucuronide
$C_{27}H_{28}O_{19}S$	688.0945	Vitexin-glucuronide-sulfate
$C_{27}H_{28}O_{20}S$	704.0895	Hydroxy-vitexin-glucuronide-sulfate
$C_{21}H_{20}O_{17}S_2$	608.0142	Hydroxy-vitexin-disulfate
$C_{33}H_{36}O_{23}$	800.1647	Hydroxy-vitexin-diglucuronide

Table S6.: Compound library of isovitexin.

Molecular formula	Exact mass	Compound
$C_{21}H_{20}O_{10}$	432.1056	Isovitexin
$C_{15}H_{10}O_5$	270.528	Apigenin
$C_7H_8O_2$	124.0524	Dihydroxy-toluene
$C_7H_6O_3$	138.0317	Hydroxy-benzoic acid
$C_8H_8O_3$	152.0473	Hydroxy-phenylacetic acid
$C_8H_8O_4$	168.0423	Dihydroxy-phenylacetic acid
$C_9H_{10}O_4$	182.0579	Hydroxy-methoxy-phenylacetic acid
$C_6H_6O_3$	126.0317	Phloroglucinol
$C_6H_6O_6S$	205.9885	Phloroglucinol-sulfate
$C_{12}H_{14}O_9$	302.0638	Phloroglucinol-glucuronide
$C_7H_8O_3$	140.0473	Methyl-phloroglucinol
$C_8H_8O_6S$	232.0042	Hydroxy-phenylacetic acid-sulfate
$C_{14}H_{16}O_9$	328.0794	Hydroxy-phenylacetic acid-glucuronide
$C_{10}H_{11}NO_4$	209.0688	Hydroxy-phenylacetic acid-glycine conjugate
$C_{11}H_{11}NO_4$	223.0845	Hydroxy-phenylpropionic acid-glycine conjugate
$C_9H_{10}O_6S$	246.0198	Hydroxy-phenylpropionic acid-sulfate
$C_{15}H_{18}O_9$	342.0951	Hydroxy-phenylpropionic acid-glucuronide
$C_{15}H_{16}O_9$	340.0794	Hydroxy-cinnamic acid-glucuronide
$C_9H_8O_6S$	244.0042	Hydroxy-cinnamic acid-sulfate
$C_9H_8O_4$	180.0423	Caffeic acid
$C_9H_8O_7S$	259.9991	Caffeic acid-sulfate
$C_{15}H_{16}O_{10}$	356.0743	Caffeic acid-glucuronide
$C_{10}H_{10}O_4$	194.0579	Ferulic acid
$C_{10}H_{10}O_7S$	274.0147	Ferulic acid-sulfate
$C_{16}H_{18}O_{10}$	370.0900	Ferulic acid-glucuronide
$C_9H_{10}O_4$	182.0597	Dihydroxy-phenylpropionic acid
$C_9H_{10}O_3$	166.0630	Hydroxy-phenylpropionic acid
$C_9H_8O_3$	164.0473	Hydroxy-phenylacrylic acid
$C_{10}H_{12}O_4$	196.0736	Hydroxy-methoxy-phenylpropionic acid
$C_{16}H_{12}O_5$	284.0685	Methoxy-apigenin
$C_{17}H_{14}O_5$	298.0841	Dimethoxy-apigenin
$C_{15}H_{10}O_6$	286.477	Hydroxy-apigenin
$C_{16}H_{12}O_6$	300.0634	Hydroxy-methoxy-apigenin
$C_{17}H_{14}O_6$	314.0790	Hydroxy-dimethoxy-apigenin
$C_{18}H_{16}O_6$	328.0947	Hydroxy-trimethoxy-apigenin
$C_{15}H_{10}O_8S$	350.0096	Apigenin-sulfate
$C_{21}H_{18}O_{11}$	446.0849	Apigenin-glucuronide
$C_{27}H_{26}O_{17}$	622.1170	Apigenin-diglucuronide
$C_{15}H_{10}O_{11}S_2$	429.9665	Apigenin-disulfate
$C_{21}H_{18}O_{14}S$	526.0417	Apigenin-glucuronide-sulfate
$C_{15}H_{10}O_9S$	366.0046	Hydroxy-apigenin-sulfate
$C_{16}H_{12}O_8S$	364.0253	Methoxy-apigenin-sulfate
$C_{16}H_{12}O_9S$	380.0202	Hydroxy-methoxy-apigenin-sulfate
$C_{22}H_{20}O_{11}$	460.1006	Methoxy-apigenin-glucuronide

$C_{21}H_{18}O_{12}$	462.0798	Hydroxy-apigenin-glucuronide
$C_{22}H_{20}O_{12}$	476.0955	Hydroxy-methoxy-apigenin-glucuronide
$C_{15}H_{10}O_{12}S_2$	445.9614	Hydroxy-apigenin-disulfate
$C_{27}H_{28}O_{18}$	638.1119	Hydroxy-apigenin-diglucuronide
$C_{21}H_{18}O_{15}S$	542.0366	Hydroxy-apigenin-glucuronide-sulfate
$C_{22}H_{22}O_{10}$	446.1213	Methoxy-isovitexin
$C_{23}H_{24}O_{10}$	460.1369	Dimethoxy-isovitexin
$C_{21}H_{20}O_{11}$	448.1006	Hydroxy-isovitexin
$C_{21}H_{20}O_{12}$	464.0955	Dihydroxy-isovitexin
$C_{22}H_{22}O_{11}$	462.1162	Hydroxy-methoxy-isovitexin
$C_{23}H_{24}O_{11}$	476.1319	Hydroxy-dimethoxy-isovitexin
$C_{22}H_{22}O_{12}$	478.1111	Dihydroxy-methoxy-isovitexin
$C_{23}H_{24}O_{12}$	492.1268	Dihydroxy-dimethoxy-isovitexin
$C_{21}H_{20}O_{13}S$	512.0625	Isovitexin-sulfate
$C_{22}H_{22}O_{13}S$	526.0781	Methoxy-isovitexin-sulfate
$C_{21}H_{20}O_{14}S$	528.0574	Hydroxy-isovitexin-sulfate
$C_{22}H_{20}O_{14}S$	542.0730	Hydroxy-methoxy-isovitexin-sulfate
$C_{21}H_{20}O_{16}S_2$	592.0193	Isovitexin-disulfate
$C_{27}H_{28}O_{16}$	608.1377	Isovitexin-glucuronide
$C_{28}H_{30}O_{16}$	622.1534	Methoxy-isovitexin-glucuronide
$C_{27}H_{28}O_{17}$	624.1326	Hydroxy-isovitexin-glucuronide
$C_{28}H_{30}O_{17}$	638.1483	Hydroxy-methoxy-isovitexin-glucuronide
$C_{33}H_{36}O_{22}$	784.1698	Isovitexin-diglucuronide
$C_{27}H_{28}O_{19}S$	688.0945	Isovitexin-glucuronide-sulfate
$C_{27}H_{28}O_{20}S$	704.0895	Hydroxy-isovitexin-glucuronide-sulfate
$C_{21}H_{20}O_{17}S_2$	608.0142	Hydroxy-isovitexin-disulfate
$C_{33}H_{36}O_{23}$	800.1647	Hydroxy-isovitexin-diglucuronide

Table S7.: Detected metabolites of orientin with an initial concentration of 10 µM and an incubation time of three hours in the Caco-2 cell metabolization assay. K: Compartment, RT: retention time, A: apical compartment, B: basolateral compartment, C: cell lysate

K	RT [min]	$[M-H]^-$ (*m/z*) found	$[M-H]^-$ (*m/z*) calculated	Molecular formula	Possible metabolite
**A**	3.146	527.0494	527.0501	$C_{21}H_{20}O_{14}S$	Orientin-sulfate
**B**	3.209	527.0500	527.0501	$C_{21}H_{20}O_{14}S$	Orientin-sulfate
**A**	8.516	477.1035	477.1038	$C_{22}H_{22}O_{12}$	Hydroxy-methoxy-orientin
**B**	8.539	477.1035	477.1038	$C_{22}H_{22}O_{12}$	Hydroxy-methoxy-orientin
**A**	9.597	461.1086	461.1089	$C_{22}H_{22}O_{11}$	Methoxy-orientin

Table S8.: Detected metabolites of orientin with an initial concentration of 100 µM and an incubation time of three hours in the Caco-2 cell metabolization assay. K: Compartment, RT: retention time, A: apical compartment, B: basolateral compartment, C: cell lysate

K	RT [min]	$[M-H]^-$ (*m/z*) found	$[M-H]^-$ (*m/z*) calculated	Molecular formula	Possible metabolite
**A**	3.204	527.0499	527.0501	$C_{21}H_{20}O_{14}S$	Orientin-sulfate
**B**	3.162	527.0493	527.0501	$C_{21}H_{20}O_{14}S$	Orientin-sulfate
**A**	4.402	463.0870	463.0882	$C_{21}H_{20}O_{12}$	Hydroxy-orientin
**A**	5.134	477.0675	477.0675	$C_{21}H_{18}O_{13}$	Hydroxy-luteolin-glucuronide
**A**	6.073	299.0559	299.0561	$C_{16}H_{12}O_6$	Methoxy-luteolin
**A**	8.497	477.1034	477.1038	$C_{22}H_{22}O_{12}$	Hydroxy-methoxy-orientin
**A**	9.578	461.1089	461.1089	$C_{22}H_{22}O_{11}$	Methoxy-orientin

Table S9.: Detected metabolites of orientin with an initial concentration of 10 µM and an incubation time of five hours in the Caco-2 cell metabolization assay. K: Compartment, RT: retention time, A: apical compartment, B: basolateral compartment, C: cell lysate

K	RT [min]	$[M-H]^-$ (*m/z*) found	$[M-H]^-$ (*m/z*) calculated	Molecular formula	Possible metabolite
**A**	3.007	527.0507	527.0501	$C_{21}H_{20}O_{14}S$	Orientin-sulfate
**A**	9.459	461.1091	461.1089	$C_{22}H_{22}O_{11}$	Methoxy-orientin

Table S10.: Detected metabolites of orientin with an initial concentration of 100 µM and an incubation time of five hours in the Caco-2 cell metabolization assay. K: Compartment, RT: retention time, A: apical compartment, B: basolateral compartment, C: cell lysate

K	RT [min]	$[M-H]^-$ (*m/z*) found	$[M-H]^-$ (*m/z*) calculated	Molecular formula	Possible metabolite
**A**	3.001	527.0501	527.0501	$C_{21}H_{20}O_{14}S$	Orientin-sulfate
**A**	4.184	463.0846	463.0882	$C_{21}H_{20}O_{12}$	Hydroxy-orientin
**A**	5.044	477.0657	477.0675	$C_{21}H_{18}O_{13}$	Hydroxy-luteolin-glucuronide
**A**	8.340	477.1029	477.1038	$C_{22}H_{22}O_{12}$	Hydroxy-methoxy-orientin
**A**	9.450	461.1085	461.1089	$C_{22}H_{22}O_{11}$	Methoxy-orientin
**C**	9.382	461.1088	461.1089	$C_{22}H_{22}O_{11}$	Methoxy-orientin

Table S11.: Detected metabolites of isoorientin with an initial concentration of 10 µM and an incubation time of three hours in the Caco-2 cell metabolization assay. K: Compartment, RT: retention time, A: apical compartment, B: basolateral compartment, C: cell lysate

K	RT [min]	$[M\text{-}H]^-$ (*m/z*) found	$[M\text{-}H]^-$ (*m/z*) calculated	Molecular formula	Possible metabolite
**A**	2.514	527.0500	527.0501	$C_{21}H_{20}O_{14}S$	Isoorientin-sulfate
**A**	5.107	527.0497	527.0501	$C_{21}H_{20}O_{14}S$	Isoorientin-sulfate
**B**	2.439	527.0500	527.0501	$C_{21}H_{20}O_{14}S$	Isoorientin-sulfate
**A**	4.044	463.0880	463.0882	$C_{21}H_{20}O_{12}$	Hydroxy-isoorientin
**A**	5.476	461.0723	461.0725	$C_{21}H_{18}O_{12}$	Luteolin-glucuronide
**A**	5.467	477.0672	477.0675	$C_{21}H_{18}O_{13}$	Hydroxy-luteolin-glucuronide
**A**	8.290	477.1034	477.1038	$C_{22}H_{22}O_{12}$	Hydroxy-methoxy-isoorientin
**B**	8.284	477.1046	477.1038	$C_{22}H_{22}O_{12}$	Hydroxy-methoxy-isoorientin
**A**	9.890	461.1089	461.1089	$C_{22}H_{22}O_{11}$	Methoxy-isoorientin
**A**	10.479	461.1089	461.1089	$C_{22}H_{22}O_{11}$	Methoxy-isoorientin

Table S12.: Detected metabolites of isoorientin with an initial concentration of 100 µM and an incubation time of three hours in the Caco-2 cell metabolization assay. K: Compartment, RT: retention time, A: apical compartment, B: basolateral compartment, C: cell lysate

K	RT [min]	$[M\text{-}H]^-$ (*m/z*) found	$[M\text{-}H]^-$ (*m/z*) calculated	Molecular formula	Possible metabolite
**A**	2.494	527.0501	527.0501	$C_{21}H_{20}O_{14}S$	Isoorientin-sulfate
**A**	5.069	527.0505	527.0501	$C_{21}H_{20}O_{14}S$	Isoorientin-sulfate
**B**	2.491	527.0498	527.0501	$C_{21}H_{20}O_{14}S$	Isoorientin-sulfate
**B**	5.008	527.0499	527.0501	$C_{21}H_{20}O_{14}S$	Isoorientin-sulfate
**C**	5.116	527.0495	527.0501	$C_{21}H_{20}O_{14}S$	Isoorientin-sulfate
**A**	4.031	463.0883	463.0882	$C_{21}H_{20}O_{12}$	Hydroxy-isoorientin
**A**	5.739	541.0654	541.0657	$C_{22}H_{22}O_{14}S$	Methoxy-isoorientin-sulfate
**A**	6.503	541.0655	541.0657	$C_{22}H_{22}O_{14}S$	Methoxy-isoorientin-sulfate
**A**	5.393	461.0723	461.0725	$C_{21}H_{18}O_{12}$	Luteolin-glucuronide
**A**	5.427	477.0674	477.0675	$C_{21}H_{18}O_{13}$	Hydroxy-luteolin-glucuronide
**A**	6.204	299.0550	299.0561	$C_{16}H_{12}O_6$	Methoxy-luteolin
**A**	8.232	477.1040	477.1038	$C_{22}H_{22}O_{12}$	Hydroxy-methoxy-isoorientin
**B**	8.252	477.1042	477.1038	$C_{22}H_{22}O_{12}$	Hydroxy-methoxy-isoorientin
**A**	9.843	461.1087	461.1089	$C_{22}H_{22}O_{11}$	Methoxy-isoorientin
**A**	10.457	461.1088	461.1089	$C_{22}H_{22}O_{11}$	Methoxy-isoorientin
**B**	10.441	461.1089	461.1089	$C_{22}H_{22}O_{11}$	Methoxy-isoorientin
**C**	10.566	461.1084	461.1089	$C_{22}H_{22}O_{11}$	Methoxy-isoorientin

Table S13.: Detected metabolites of isoorientin with an initial concentration of 10 µM and an incubation time of five hours in the Caco-2 cell metabolization assay. K: Compartment, RT: retention time, A: apical compartment, B: basolateral compartment, C: cell lysate

K	RT [min]	$[M\text{-}H]^-$ (*m/z*) found	$[M\text{-}H]^-$ (*m/z*) calculated	Molecular formula	Possible metabolite
**A**	2.332	527.0505	527.0501	$C_{21}H_{20}O_{14}S$	Isoorientin-sulfate
**A**	4.673	527.0507	527.0501	$C_{21}H_{20}O_{14}S$	Isoorientin-sulfate
**A**	6.221	477.1034	477.1038	$C_{22}H_{22}O_{12}$	Hydroxy-methoxy-isoorientin
**A**	9.474	461.1085	461.1089	$C_{22}H_{22}O_{11}$	Methoxy-isoorientin
**A**	10.079	461.1080	461.1089	$C_{22}H_{22}O_{11}$	Methoxy-isoorientin

Table S14.: Detected metabolites of isoorientin with an initial concentration of 100 µM and an incubation time of five hours in the Caco-2 cell metabolization assay. K: Compartment, RT: retention time, A: apical compartment, B: basolateral compartment, C: cell lysate

K	RT [min]	$[M\text{-}H]^-$ (*m/z*) found	$[M\text{-}H]^-$ (*m/z*) calculated	Molecular formula	Possible metabolite
**A**	2.308	527.0493	527.0501	$C_{21}H_{20}O_{14}S$	Isoorientin-sulfate
**A**	4.644	527.0500	527.0501	$C_{21}H_{20}O_{14}S$	Isoorientin-sulfate
**B**	2.296	527.0496	527.0501	$C_{21}H_{20}O_{14}S$	Isoorientin-sulfate
**B**	4.682	527.0500	527.0501	$C_{21}H_{20}O_{14}S$	Isoorientin-sulfate
**C**	4.692	527.0521	527.0501	$C_{21}H_{20}O_{14}S$	Isoorientin-sulfate
**A**	4.178	463.0885	463.0882	$C_{21}H_{20}O_{12}$	Hydroxy-isoorientin
**A**	5.206	541.0654	541.0657	$C_{21}H_{22}O_{14}S$	Methoxy-isoorientin-sulfate
**A**	5.972	541.0649	541.0657	$C_{21}H_{22}O_{14}S$	Methoxy-isoorientin-sulfate
**A**	5.181	461.0717	461.0725	$C_{21}H_{18}O_{12}$	Luteolin-glucuronide
**A**	5.232	477.0665	477.0675	$C_{21}H_{18}O_{13}$	Hydroxy-luteolin-glucuronide
**A**	9.496	461.1089	461.1089	$C_{22}H_{22}O_{11}$	Methoxy-isoorientin
**A**	10.106	461.1090	461.1089	$C_{22}H_{22}O_{11}$	Methoxy-isoorientin
**C**	10.178	461.1107	461.1089	$C_{22}H_{22}O_{11}$	Methoxy-isoorientin

Table S15.: Detected metabolites of schaftoside with an initial concentration of 10 µM and an incubation time of three hours in the Caco-2 cell metabolization assay. K: Compartment, RT: retention time, A: apical compartment, B: basolateral compartment, C: cell lysate

K	RT [min]	$[M\text{-}H]^-$ (*m/z*) found	$[M\text{-}H]^-$ (*m/z*) calculated	Molecular formula	Possible metabolite
**A**	7.732	593.1509	593.1512	$C_{27}H_{30}O_{15}$	Hydroxy-methoxy-schaftoside

Table S16.: Detected metabolites of schaftoside with an initial concentration of 100 µM and an incubation time of three hours in the Caco-2 cell metabolization assay. K: Compartment, RT: retention time, A: apical compartment, B: basolateral compartment, C: cell lysate

K	RT [min]	$[M\text{-}H]^-$ (*m/z*) found	$[M\text{-}H]^-$ (*m/z*) calculated	Molecular formula	Possible metabolite
**A**	5.804	579.1350	579.1355	$C_{26}H_{28}O_{15}$	Hydroxy-schaftoside
**A**	7.716	593.1506	593.1512	$C_{27}H_{30}O_{15}$	Hydroxy-methoxy-schaftoside
**B**	7.744	593.1507	593.1512	$C_{27}H_{30}O_{15}$	Hydroxy-methoxy-schaftoside

Table S17.: Detected metabolites of schaftoside with an initial concentration of 10 µM and an incubation time of five hours in the Caco-2 cell metabolization assay. K: Compartment, RT: retention time, A: apical compartment, B: basolateral compartment, C: cell lysate

K	RT [min]	$[M\text{-}H]^-$ (*m/z*) found	$[M\text{-}H]^-$ (*m/z*) calculated	Molecular formula	Possible metabolite
**A**	7.383	593.1513	593.1512	$C_{27}H_{30}O_{15}$	Hydroxy-methoxy-schaftoside

Table S18.: Detected metabolites of schaftoside with an initial concentration of 100 µM and an incubation time of five hours in the Caco-2 cell metabolization assay. K: Compartment, RT: retention time, A: apical compartment, B: basolateral compartment, C: cell lysate

K	RT [min]	$[M\text{-}H]^-$ (*m/z*) found	$[M\text{-}H]^-$ (*m/z*) calculated	Molecular formula	Possible metabolite
**A**	5.524	579.1349	579.1355	$C_{26}H_{28}O_{15}$	Hydroxy-schaftoside
**A**	7.549	593.1512	593.1512	$C_{27}H_{30}O_{15}$	Hydroxy-methoxy-schaftoside

Table S19.: Detected metabolites of isoschaftoside with an initial concentration of 10 µM and an incubation time of three hours in the Caco-2 cell metabolization assay. K: Compartment, RT: retention time, A: apical compartment, B: basolateral compartment, C: cell lysate

K	RT [min]	$[M\text{-}H]^-$ (*m/z*) found	$[M\text{-}H]^-$ (*m/z*) calculated	Molecular formula	Possible metabolite
**A**	8.720	593.1511	593.1512	$C_{27}H_{30}O_{15}$	Hydroxy-methoxy-isoschaftoside

Table S20.: Detected metabolites of isoschaftoside with an initial concentration of 100 µM and an incubation time of three hours in the Caco-2 cell metabolization assay. K: Compartment, RT: retention time, A: apical compartment, B: basolateral compartment, C: cell lysate

K	RT [min]	[M-H]$^-$ (*m/z*) found	[M-H]$^-$ (*m/z*) calculated	Molecular formula	Possible metabolite
**A**	8.705	593.1499	593.1512	$C_{27}H_{30}O_{15}$	Hydroxy-methoxy-isoschaftoside
**B**	8.710	593.1513	593.1512	$C_{27}H_{30}O_{15}$	Hydroxy-methoxy-isoschaftoside
**A**	10.712	577.1565	577.1563	$C_{27}H_{30}O_{14}$	Methoxy-isoschaftoside
**A**	11.597	607.1668	607.1668	$C_{28}H_{32}O_{15}$	Hydroxy-dimethoxy-isoschaftoside

Table S21.: Detected metabolites of isoschaftoside with an initial concentration of 100 µM and an incubation time of five hours in the Caco-2 cell metabolization assay. K: Compartment, RT: retention time, A: apical compartment, B: basolateral compartment, C: cell lysate

K	RT [min]	[M-H]$^-$ (*m/z*) found	[M-H]$^-$ (*m/z*) calculated	Molecular formula	Possible metabolite
**A**	8.469	593.1456	593.1512	$C_{27}H_{30}O_{15}$	Hydroxy-methoxy-isoschaftoside
**A**	11.464	607.1668	607.1668	$C_{28}H_{32}O_{15}$	Hydroxy-dimethoxy-isoschaftoside

Table S22.: Detected metabolites of vitexin with an initial concentration of 100 µM and an incubation time of three hours in the Caco-2 cell metabolization assay. K: Compartment, RT: retention time, A: apical compartment, B: basolateral compartment, C: cell lysate

K	RT [min]	[M-H]$^-$ (*m/z*) found	[M-H]$^-$ (*m/z*) calculated	Molecular formula	Possible metabolite
**A**	9.615	461.1091	461.1089	$C_{22}H_{22}O_{11}$	Hydroxy-methoxy-vitexin
**B**	9.646	461.1092	461.1089	$C_{22}H_{22}O_{11}$	Hydroxy-methoxy-vitexin

Table S23.: Detected metabolites of vitexin with an initial concentration of 100 µM and an incubation time of five hours in the Caco-2 cell metabolization assay. K: Compartment, RT: retention time, A: apical compartment, B: basolateral compartment, C: cell lysate

K	RT [min]	[M-H]$^-$ (*m/z*) found	[M-H]$^-$ (*m/z*) calculated	Molecular formula	Possible metabolite
**A**	5.695	447.0934	447.0933	$C_{21}H_{20}O_{11}$	Hydroxy-vitexin

Table S24.: Detected metabolites of isovitexin with an initial concentration of 10 µM and an incubation time of three hours in the Caco-2 cell metabolization assay. K: Compartment, RT: retention time, A: apical compartment, B: basolateral compartment, C: cell lysate

K	RT [min]	[M-H]$^-$ (*m/z*) found	[M-H]$^-$ (*m/z*) calculated	Molecular formula	Possible metabolite
**A**	8.491	447.0934	447.0933	$C_{21}H_{20}O_{11}$	Hydroxy-isovitexin
**A**	8.912	445.1140	445.1140	$C_{22}H_{22}O_{10}$	Methoxy-isovitexin
**A**	9.468	461.1093	461.1089	$C_{22}H_{22}O_{11}$	Hydroxy-methoxy-isovitexin
**A**	11.658	364.9978	364.9973	$C_{15}H_{10}O_9S$	Hydroxy-apigenin-sulfate
**A**	11.909	379.0133	379.0129	$C_{16}H_{12}O_9S$	Hydroxy-methoxy-apigenin-sulfate
**A**	11.910	299.0564	299.0561	$C_{16}H_{12}O_6$	Hydroxy-methoxy-apigenin

Table S25.: Detected metabolites of isovitexin with an initial concentration of 100 µM and an incubation time of three hours in the Caco-2 cell metabolization assay. K: Compartment, RT: retention time, A: apical compartment, B: basolateral compartment, C: cell lysate

K	RT [min]	[M-H]$^-$ (*m/z*) found	[M-H]$^-$ (*m/z*) calculated	Molecular formula	Possible metabolite
**A**	8.501	447.0928	447.0933	$C_{21}H_{20}O_{11}$	Hydroxy-isovitexin
**A**	8.905	445.1144	445.1140	$C_{21}H_{22}O_{10}$	Methoxy-isovitexin
**A**	9.451	461.1098	461.1089	$C_{22}H_{22}O_{11}$	Hydroxy-methoxy-isovitexin
**A**	11.648	364.9978	364.9973	$C_{15}H_{10}O_9S$	Hydroxy-apigenin-sulfate
**A**	11.651	475.1251	475.1246	$C_{23}H_{24}O_{11}$	Hydroxy-dimethoxy-isovitexin
**B**	11.654	475.1248	475.1246	$C_{23}H_{24}O_{11}$	Hydroxy-dimethoxy-isovitexin
**A**	11.900	299.0562	299.0561	$C_{16}H_{12}O_6$	Hydroxy-methoxy-apigenin
**A**	11.957	379.0135	379.0129	$C_{16}H_{12}O_9S$	Hydroxy-methoxy-apigenin-sulfate
**B**	11.903	379.0131	379.0129	$C_{16}H_{12}O_9S$	Hydroxy-methoxy-apigenin-sulfate

Table S26.: Detected metabolites of isovitexin with an initial concentration of 10 µM and an incubation time of five hours in the Caco-2 cell metabolization assay. K: Compartment, RT: retention time, A: apical compartment, B: basolateral compartment, C: cell lysate

K	RT [min]	[M-H]$^-$ (*m/z*) found	[M-H]$^-$ (*m/z*) calculated	Molecular formula	Possible metabolite
**A**	8.569	447.0937	447.0933	$C_{21}H_{20}O_{11}$	Hydroxy-isovitexin
**A**	9.564	461.1093	461.1089	$C_{22}H_{22}O_{11}$	Hydroxy-methoxy-isovitexin
**A**	11.681	475.1248	475.1246	$C_{23}H_{24}O_{11}$	Hydroxy-dimethoxy-isovitexin
**A**	11.702	364.9976	364.9973	$C_{15}H_{10}O_9S$	Hydroxy-apigenin-sulfate
**A**	11.953	379.0133	379.0129	$C_{16}H_{12}O_9S$	Hydroxy-methoxy-apigenin-sulfate

Table S27.: Detected metabolites of isovitexin with an initial concentration of 100 µM and an incubation time of five hours in the Caco-2 cell metabolization assay. K: Compartment, RT: retention time, A: apical compartment, B: basolateral compartment, C: cell lysate

K	RT [min]	[M-H]$^-$ (*m/z*) found	[M-H]$^-$ (*m/z*) calculated	Molecular formula	Possible metabolite
**A**	8.514	447.0932	447.0933	$C_{21}H_{20}O_{11}$	Hydroxy-isovitexin
**A**	8.951	445.1145	445.1140	$C_{21}H_{22}O_{10}$	Methoxy-isovitexin
**A**	9.478	461.1099	461.1089	$C_{22}H_{22}O_{11}$	Hydroxy-methoxy-isovitexin
**A**	11.655	364.9984	364.9973	$C_{15}H_{10}O_9S$	Hydroxy-apigenin-sulfate
**A**	11.656	475.1252	475.1246	$C_{23}H_{24}O_{11}$	Hydroxy-dimethoxy-isovitexin
**A**	11.906	299.0566	299.0561	$C_{16}H_{12}O_6$	Hydroxy-methoxy-apigenin
**A**	11.960	379.0131	379.0129	$C_{16}H_{12}O_9S$	Hydroxy-methoxy-apigenin-sulfate
**B**	11.908	379.0132	379.0129	$C_{16}H_{12}O_9S$	Hydroxy-methoxy-apigenin-sulfate
